# Characterizing clinically recognized hypertrophic cardiomyopathy in six European countries using real-world data

**DOI:** 10.1093/eschf/xvag218

**Published:** 2026-08-20

**Authors:** Marta Alcalde-Herraiz, Antonella Delmestri, Hezekiah Omulo, Elvira Bräuner, Susanne Bruun, Raeleesha Norris, Annika Vivirito, Alexander Harms, Jakov Vuković, Ivan Pristaš, Anamaria Jurčević, Marko Čavlina, Antea Jezidžić, Pero Ivanko, Saeed Hayati, Nhung T H Trinh, Hedvig Marie Egeland Nordeng, Talita Duarte-Salles, Anna Palomar-Cros, Agustina Giuliodori, Antonio Gómez-Outes, Patrick Vrijlandt, María Clara Restrepo-Méndez, Edward Burn, Albert Prats-Uribe, Anna Saura-Lázaro

**Affiliations:** Health Data Sciences, Botnar Research Centre, Nuffield Department of Orthopaedics, Rheumatology, and Musculoskeletal Sciences, University of Oxford, OX3 7LD Oxford, England; Health Data Sciences, Botnar Research Centre, Nuffield Department of Orthopaedics, Rheumatology, and Musculoskeletal Sciences, University of Oxford, OX3 7LD Oxford, England; Health Data Sciences, Botnar Research Centre, Nuffield Department of Orthopaedics, Rheumatology, and Musculoskeletal Sciences, University of Oxford, OX3 7LD Oxford, England; Data Analytics Center, Danish Medicines Agency, Copenhagen, Denmark; Data Analytics Center, Danish Medicines Agency, Copenhagen, Denmark; Data Science/Health Services Research, InGef—Institute for Applied Health Research Berlin GmbH, Berlin, Germany; Data Science/Health Services Research, InGef—Institute for Applied Health Research Berlin GmbH, Berlin, Germany; Data Science/Health Services Research, InGef—Institute for Applied Health Research Berlin GmbH, Berlin, Germany; Medical Informatics and Biostatistics Division, Croatian National Institute of Public Health, Zagreb, Croatia; Medical Informatics and Biostatistics Division, Croatian National Institute of Public Health, Zagreb, Croatia; Medical Informatics and Biostatistics Division, Croatian National Institute of Public Health, Zagreb, Croatia; Medical Informatics and Biostatistics Division, Croatian National Institute of Public Health, Zagreb, Croatia; Medical Informatics and Biostatistics Division, Croatian National Institute of Public Health, Zagreb, Croatia; Medical Informatics and Biostatistics Division, Croatian National Institute of Public Health, Zagreb, Croatia; PharmacoEpidemiology and Drug Safety Research Group, Department of Pharmacy, and PharmaTox Strategic Research Initiative, University of Oslo, Oslo, Norway; PharmacoEpidemiology and Drug Safety Research Group, Department of Pharmacy, and PharmaTox Strategic Research Initiative, University of Oslo, Oslo, Norway; PharmacoEpidemiology and Drug Safety Research Group, Department of Pharmacy, and PharmaTox Strategic Research Initiative, University of Oslo, Oslo, Norway; Department of Child Health and Development, Norwegian Institute of Public Health, Oslo, Norway; Unitat Transversal de Recerca (UTR), Fundació Institut Universitari Per a la Recerca a l'Atenció Primària de Salut Jordi Gol i Gurina (IDIAPJGol), Barcelona, Spain; Department of Medical Informatics, Erasmus University Medical Center, Rotterdam, The Netherlands; Unitat Transversal de Recerca (UTR), Fundació Institut Universitari Per a la Recerca a l'Atenció Primària de Salut Jordi Gol i Gurina (IDIAPJGol), Barcelona, Spain; Unitat Transversal de Recerca (UTR), Fundació Institut Universitari Per a la Recerca a l'Atenció Primària de Salut Jordi Gol i Gurina (IDIAPJGol), Barcelona, Spain; Division of Pharmacology and Clinical Drug Evaluation, Medicines for Human Use, Spanish Agency for Medicines and Healthcare Products (AEMPS), Madrid, Spain; Dutch Medicines Evaluation Board, Utrecht, The Netherlands; Deparment of Clinical Pharmacy and Pharmacology, University of Groningen, University Medical Centre Groningen, Groningen, The Netherlands; European Medicines Agency, Amsterdam, The Netherlands; Health Data Sciences, Botnar Research Centre, Nuffield Department of Orthopaedics, Rheumatology, and Musculoskeletal Sciences, University of Oxford, OX3 7LD Oxford, England; Health Data Sciences, Botnar Research Centre, Nuffield Department of Orthopaedics, Rheumatology, and Musculoskeletal Sciences, University of Oxford, OX3 7LD Oxford, England; Health Data Sciences, Botnar Research Centre, Nuffield Department of Orthopaedics, Rheumatology, and Musculoskeletal Sciences, University of Oxford, OX3 7LD Oxford, England

**Keywords:** Hypertrophic cardiomyopathy, Obstructive hypertrophic cardiomyopathy, Observational studies, Prevalence, Real-world Data, Europe

## Abstract

**Background and aims:**

Hypertrophic cardiomyopathy (HCM) is the most common inherited cardiomyopathy. However, large-scale epidemiological evidence remains scarce due to challenges in real-world disease recognition. This study aimed to characterize clinically recognized HCM and obstructive HCM (oHCM) across six European countries regarding prevalence, demographics, and clinical characteristics.

**Methods:**

We conducted a retrospective cohort study using routinely collected healthcare data from six European countries, all mapped to the Observational Medical Outcomes Partnership (OMOP) Common Data Model within the DARWIN EU® network: CPRD-GOLD (UK), DK-DHR (Denmark), InGef RDB (Germany), NAJS (Croatia), NLHR (Norway), and SIDIAP (Spain). Clinically recognized HCM was defined based on recorded diagnoses captured in routine healthcare data, rather than on imaging-, haemodynamic-, or genotype-confirmed population screening. Adults (≥18 years) with a first recorded HCM or oHCM diagnosis after 2010 were included. We estimated annual period prevalence and described recorded comorbidities, diagnostic measurements, and treatments before, at, and after diagnosis.

**Results:**

Among 40 277 individuals with HCM, 12 363 (31%) were first diagnosed with oHCM. Females were older than males at diagnosis (median 67–78 vs 57–68 years). Annual period prevalence increased over time, ranging from 0.04% (95% Confidence interval: 0.04–0.05) to 0.24% (0.23–0.24) in recent years. Prevalence was higher in males, but differences diminished among those aged ≥80 and with oHCM. Cardiovascular comorbidities were frequently recorded before and at the time of first diagnosis, especially hypertension, cardiac arrhythmias, ischaemic heart disease, and heart failure. Beta-blockers, diuretics, and angiotensin-converting enzyme inhibitors were the most common treatments. Most comorbidities and treatments were recorded over a year before HCM diagnosis.

**Conclusions:**

An increase in the prevalence of clinically recognized HCM across Europe was observed over time. This trend is likely multifactorial and may reflect changes in disease recognition, clinical practice, demographics, database-related factors, and potentially the underlying disease burden. Cardiovascular comorbidities and treatments were frequently recorded prior to diagnosis, which may suggest that some patients would benefit from increased diagnostic awareness.

## Graphical Abstract

**Graphical Abstract:**
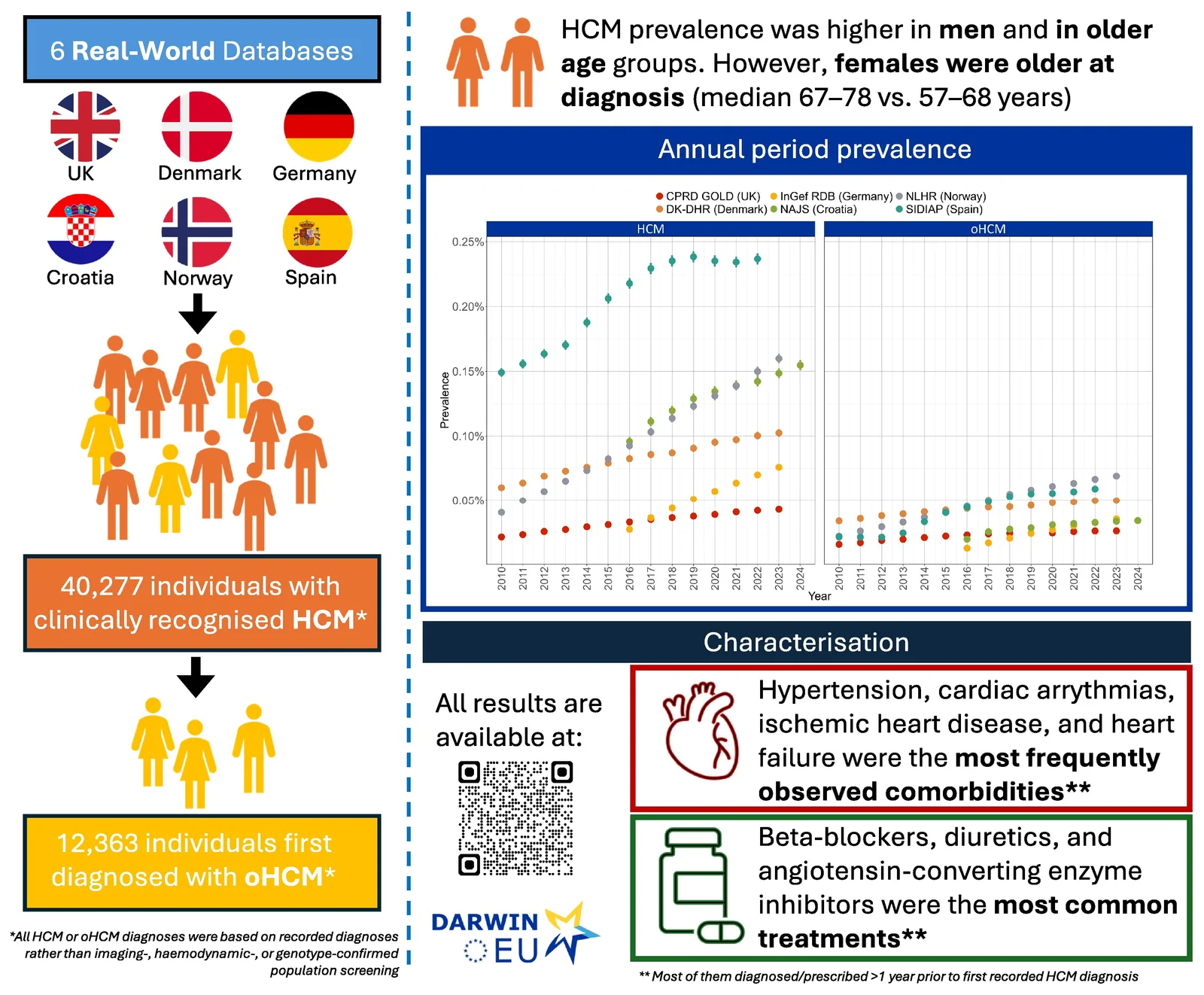

## Introduction

Hypertrophic cardiomyopathy (HCM) is characterized by increased wall thickness or mass of the left ventricle. A diagnosis of HCM requires this hypertrophy to be present in the absence of any other cardiac, metabolic, or systemic disease that could cause it.^1^ HCM is classified into two types based on the presence or absence of left ventricular outflow tract (LVOT) obstruction. The obstructive form of HCM (oHCM) is observed in approximately 66% of patients^2,3^ and is associated with a higher likelihood of developing clinical symptoms compared to the non-obstructive HCM.^4,5^

Patients with HCM have an increased risk of cardiovascular complications, including atrial fibrillation (AF), stroke, heart failure (HF), and sudden cardiac death (SCD).^1,6,7^ Pharmacological treatments for symptomatic relief typically include beta blockers as first-line therapies, followed by non-dihydropyridine calcium-channel blockers, or disopyramide. In 2022, mavacamten, the first targeted treatment for symptomatic oHCM, was approved in the USA,^8^ and the European Medicines Agency (EMA) recommended marketing authorization for use in Europe the following year.^9–11^ Surgical interventions, such as alcohol septal ablation and septal myectomy, or implantable cardioverter defibrillators or pacemakers, are also available for patients with severe oHCM, although they do not address the underlying myocardial pathology.

Despite HCM being the most common inherited cardiomyopathy and a major contributor to cardiovascular morbidity and mortality across all age groups, estimating its prevalence has proven to be challenging. Factors such as the relative rarity of the condition, the high proportion of asymptomatic patients, and diagnostic difficulties (as it can be easily mistaken for other conditions also presenting as hypertrophy of the left ventricle) can complicate accurate estimation.^12^ Furthermore, fragmentation across healthcare databases can hinder accurate estimation, as patient follow-up may be incomplete. As a result, large-scale epidemiological studies on the demographics and morbidity burden of HCM in Europe are scarce, with many existing studies relying solely on inpatient records that do not capture the full extent of the disease burden.

This study aimed to address some of these gaps (e.g. incomplete follow-up) by characterizing clinically recognized HCM and oHCM, separately, on a large scale across six European countries. Specifically, we estimated the annual prevalence of clinically recognized HCM and oHCM and described the frequency of predefined comorbidities, diagnostic measurements, and HCM-related treatments before, at the time of, and after the first recorded HCM or oHCM diagnosis.

## Methods

### Study design, population, and period

We performed a retrospective cohort study of individuals aged 18 years or older at the time of their first recorded diagnosis of HCM or oHCM (defined as the index date). This represents the earliest documented diagnosis within the available data and should not be interpreted as the true clinical onset of HCM. Participant identification started from 1 January 2010, or, for databases with later availability, from the date data became accessible, and continued until the end of available data in each data source (Supplementary Table S1). All individuals were required to have at least one year of continuous observation prior to index date. Individuals with a recorded HCM or oHCM diagnosis before their registration in the database were excluded. Supplementary Figure S1 shows the HARPER Diagram^13^ of the study design.

Follow-up started on the index date and continued until the earliest of the following events: (i) loss to follow-up, (ii) date of death, or (iii) the end of the database observation period. For individuals with multiple observation periods in a database, only the first period was considered for analysis.

The study protocol is available at the HMA-EMA Catalogues of Real-World Data sources and studies webpage.^14^ The study code is publicly available on GitHub: https://github.com/darwin-eu-studies/EUPAS1000000430.

### Data sources

We used six routinely collected healthcare data sources from Europe, all mapped to the Observational Medical Outcomes Partnership (OMOP) Common Data Model and onboarded to the European Medicines Agency-funded Data Analysis and Real World Interrogation Network (DARWIN EU®)^15^: two large primary care data sources (Clinical Practice Research Datalink (CPRD) GOLD, United Kingdom (UK) and the Information System for the Development of Research in Primary Care (SIDIAP), Catalonia, Spain),^9^ with the latter also being linked to hospital discharge data; two Nordic nationwide registries that include both primary and secondary/hospital data (Danish Data Health Registries (DK-DHR), Denmark and Norwegian Linked Health Registry data (NLHR), Norway); and two nationwide claims databases (InGef Research Database (InGef RDB), Germany and Croatian National Public Health Information System (NAJS), Croatia), with NAJS including both primary and secondary/hospital data, while InGef RDB included hospital data only in this study. Please refer to Supplementary Table S1 for a summary of the data captured by each database.

### Variables

The study outcomes, HCM and oHCM, were identified using SNOMED condition codes (Supplementary Note S1). Only clinically recognized HCM or oHCM diagnoses that led to a healthcare encounter and generated a recorded diagnosis were therefore captured; thus, the study reflects clinically recognized HCM identified through routine healthcare data rather than imaging-, haemodynamic-, or genotype-confirmed population screening. Conditions known to mimic genetic forms of HCM, including mitochondrial diseases, Danon disease, Friedreich’s ataxia, Leopard syndrome, Noonan syndrome, Anderson–Fabry disease, and amyloidosis, were excluded from the code list. HCM and oHCM phenotypes were developed using the standardized and reproducible DARWIN EU® phenotyping framework, involving systematic code list development, iterative review by clinical experts and epidemiologists, and cohort diagnostics to enhance the transparency, reproducibility, and clinical relevance of the phenotype definitions.^16^

Based on clinical expertise and existing literature, we predefined 19 comorbidities, 12 diagnostic measurements, 15 medications, and five treatment procedures relevant to HCM and oHCM (Supplementary Note S1).

### Statistical methods

#### Annual prevalence

We estimated the annual period prevalence for clinically recognized HCM and oHCM separately, from 1 January to 31 December for each calendar year. Annual prevalence was calculated by dividing the number of individuals with a recorded diagnosis of HCM or oHCM (numerator) by the total active population within the respective calendar year (denominator). We assumed diagnosis persistence from first occurrence until the end of follow-up. To be included in the numerator, participants needed a recorded diagnosis of HCM. For the denominator, a minimum of 365 days of prior observation and a day of observation within the annual period was required to be considered active population.

Prevalence estimates were stratified by sex and age group, noting that individuals could contribute to multiple age groups. The 95% confidence intervals (CI) were estimated using the Wilson method. Further details on age group definitions are provided in Supplementary Note S1. To enable a meaningful comparison across databases with different age structures, we estimated directly age-standardized prevalence using the World Health Organization single-age world standard population^17^ as the reference. For the age standardized estimates, the 95% CI were calculated using the gamma distribution, which is considered more suitable for generating accurate CI when events are rare or counts are low. See Supplementary Note S2 for more details.

#### Patient-level characterization

Patient-level characterization was performed across different time windows relative to the index date (Supplementary Note S1) and stratified by sex. For each time window, we assessed the number and percentage of individuals with predefined comorbidities, diagnostic measurements, medications, and treatment procedures. Both new and ongoing medication use was included. Prescription durations were derived from the drug exposure table. Drug episodes were merged if gaps were ≤90 days. Only patients under observation during each specific window were included in the denominator. We assumed missing records indicated the absence of the respective comorbidity, diagnostic measurement, medication prescription, or treatment procedure for the individual. Therefore, this analysis estimated the recorded frequency of these variables within each database rather than their true clinical occurrence. DK-DHR and NLHR did not have measurement data.

Additionally, we calculated the median and interquartile range (IQR) of the time from the first occurrence of the predefined covariates to the first recorded HCM or oHCM diagnosis.

Cell counts fewer than five were suppressed to preserve confidentiality. All analyses were performed with R (Version 4.4.1). The following packages were used for data manipulation and analysis: dplyr (v1.1.4),^18^ CDMConnector (v1.7.0),^19^ CodelistGenerator(v3.3.1),^20^ CohortConstructor (v0.3.4),^21^ IncidencePrevalence (v1.0.0),^22^ CohortCharacteristics (v0.4.0),^23^ visOmopResults (v1.0.0).^24^

## Results

Main results are presented here for clarity. All results can be explored in an interactive web application (available from: https://data-dev.darwin-eu.org/HCM_Characterisation_Manuscript/). The full study report is accessible at the HMA-EMA Catalogues of Real-World Data sources and studies webpage.^25^

### Study participants

We identified 40 277 individuals with a first recorded diagnosis of HCM across all study databases (Supplementary Figure S2): 1780 individuals in CPRD GOLD; 4826 in DK-DHR; 6311 in InGef RDB; 6653 in NAJS; 7114 in NLHR; and 13 593 in SIDIAP. Among them, oHCM cases accounted for 1062 (60%) in CPRD GOLD; 1934 (40%) in DK-DHR; 2551 (40%) in InGef RDB; 1559 (23%) in NAJS; 2413 (34%) in NLHR; and 2844 (21%) in SIDIAP.

In the clinically recognized HCM cohort, the proportion of males ranged from 52% to 63% (NAJS—CPRD GOLD) (*Table 1*), with median ages at first recorded diagnosis of 57–68 years (CPRD GOLD—SIDIAP) for males and 67–78 years (CPRD GOLD—SIDIAP) for females. In the clinically recognized oHCM cohort, males accounted for 43%–61% (SIDIAP—CPRD GOLD, InGef RDB), with median ages at first recorded diagnosis of 57–68 years (CPRD GOLD—SIDIAP) for males and 67–76 (CPRD GOLD—SIDIAP, NAJS) for females.

**Table 1:** Baseline characteristics of study participants by clinically recognized hypertrophic cardiomyopathy (HCM) and obstructive HCM (oHCM) cohorts, and study databases. The frequency of other comorbidities (aberrant premature complexes, atrioventricular block, cardiac arrhythmia, coronary arteriosclerosis, premature atrial contraction, sudden cardiac death, sustained ventricular tachycardia, and ventricular fibrillation) any time prior to the first recorded HCM or oHCM recorded diagnosis can be found in the interactive web application

Variables	Levels	Statistics	Database (Country)											
			CPRD GOLD (UK)		DK-DHR (Denmark)		InGef RDB (Germany)		NAJS (Croatia)		NLHR (Norway)		SIDIAP (Spain)	
			Cohort name											
			HCM	oHCM	HCM	oHCM	HCM	oHCM	HCM	oHCM	HCM	oHCM	HCM	oHCM
Baseline characteristics														
Number of subjects	—	*N*	1780	1062	4826	1934	6311	2551	6653	1559	7114	2413	13 593	2844
Age (years)	—	Median [Q25-Q75]	61 [50–71]	61 [50–71]	67 [56–77]	69 [60–77]	66 [54–78]	66 [55–78]	71 [60–79]	72 [63–80]	66 [54–75]	67 [58–75]	73 [61–82]	72 [61–81]
		Range [Min-Max]	18–95	18–95	18–101	18–99	18–103	18–100	18–103	19–100	18–99	18–97	18–101	18–101
Sex	Female	*N* (%)	663 (37)	417 (39)	2272 (47)	1077 (56)	2431 (39)	1167 (46)	3170 (48)	772 (50)	2861 (40)	1163 (48)	6159 (45)	1620 (57)
	Male	*N* (%)	1117 (63)	645 (61)	2554 (53)	857 (44)	3880 (61)	1384 (54)	3483 (52)	787 (50)	4253 (60)	1250 (52)	7434 (55)	1224 (43)
Comorbidities any time prior to first recorded HCM or oHCM recorded diagnosis														
Atrial fibrillation	—	*N* (%)	191 (11)	111 (10)	241 (5)	73 (4)	1194 (19)	354 (14)	642 (10)	177 (11)	850 (12)	216 (9)	3325 (24)	525 (18)
Chronic kidney disease	—	*N* (%)	197 (11)	114 (11)	217 (4)	58 (3)	1066 (17)	330 (13)	700 (11)	180 (12)	380 (5)	136 (6)	3327 (24)	566 (20)
Chronic obstructive pulmonary disease	—	*N* (%)	92 (5)	62 (6)	366 (8)	158 (8)	494 (8)	190 (7)	1113 (17)	314 (20)	681 (10)	250 (10)	2075 (15)	327 (11)
Disorder of lipoprotein and/or lipid metabolism	—	*N* (%)	198 (11)	119 (11)	754 (16)	299 (15)	1574 (25)	558 (22)	2711 (41)	657 (42)	1481 (21)	527 (22)	6177 (45)	1268 (45)
Essential hypertension	—	*N* (%)	555 (31)	344 (32)	93 (2)	28 (1)	3081 (49)	1151 (45)	5487 (82)	1345 (86)	4067 (57)	1483 (61)	8101 (60)	1619 (57)
Heart failure	—	*N* (%)	79 (4)	46 (4)	669 (14)	210 (11)	1623 (26)	522 (20)	1368 (21)	361 (23)	1695 (24)	503 (21)	4051 (30)	632 (22)
Ischaemic stroke	—	*N* (%)	84 (5)	56 (5)	474 (10)	176 (9)	450 (7)	165 (6)	601 (9)	172 (11)	796 (11)	272 (11)	1374 (10)	241 (8)
Ischaemic heart disease	—	*N* (%)	258 (14)	161 (15)	1253 (26)	508 (26)	1763 (28)	590 (23)	2136 (32)	570 (37)	2103 (30)	724 (30)	2950 (22)	530 (19)
Obesity	—	*N* (%)	54 (3)	35 (3)	368 (8)	148 (8)	778 (12)	272 (11)	746 (11)	186 (12)	501 (7)	157 (7)	3826 (28)	783 (28)
Type 2 diabetes mellitus	—	*N* (%)	159 (9)	101 (10)	490 (10)	182 (9)	1004 (16)	339 (13)	2087 (31)	506 (32)	747 (11)	270 (11)	3750 (28)	632 (22)
Valvular heart disease	—	*N* (%)	112 (6)	66 (6)	628 (13)	379 (20)	927 (15)	361 (14)	947 (14)	299 (19)	1515 (21)	673 (28)	3067 (23)	699 (25)

### Prevalence of clinically recognized HCM and oHCM

The prevalence of first recorded clinically recognized HCM increased over time across all study databases, with variations in estimates (*Figure 1*, Supplementary Table S2). CPRD GOLD reported the lowest prevalence, reaching 0.043% (95% CI: 0.041%–0.046%) in 2023. This was followed by InGef RDB, which peaked at 0.076% (95% CI: 0.074%–0.078%) in 2023, DK-DHR, which reported a maximum of 0.102% (95% CI: 0.100%–0.105%) in 2023, NAJS, which rose to 0.155% (95% CI: 0.151%–0.159%) in 2024, and by NLHR, which attained 0.160% (95% CI: 0.156%–0.164%) in 2023. The highest prevalence was observed in SIDIAP, reaching 0.237% (95%CI: 0.233% to 0.241%) in 2019 before declining slightly in 2020–2021. Prevalence patterns persisted after age standardization (Supplementary Figure S3).

**Figure 1:**
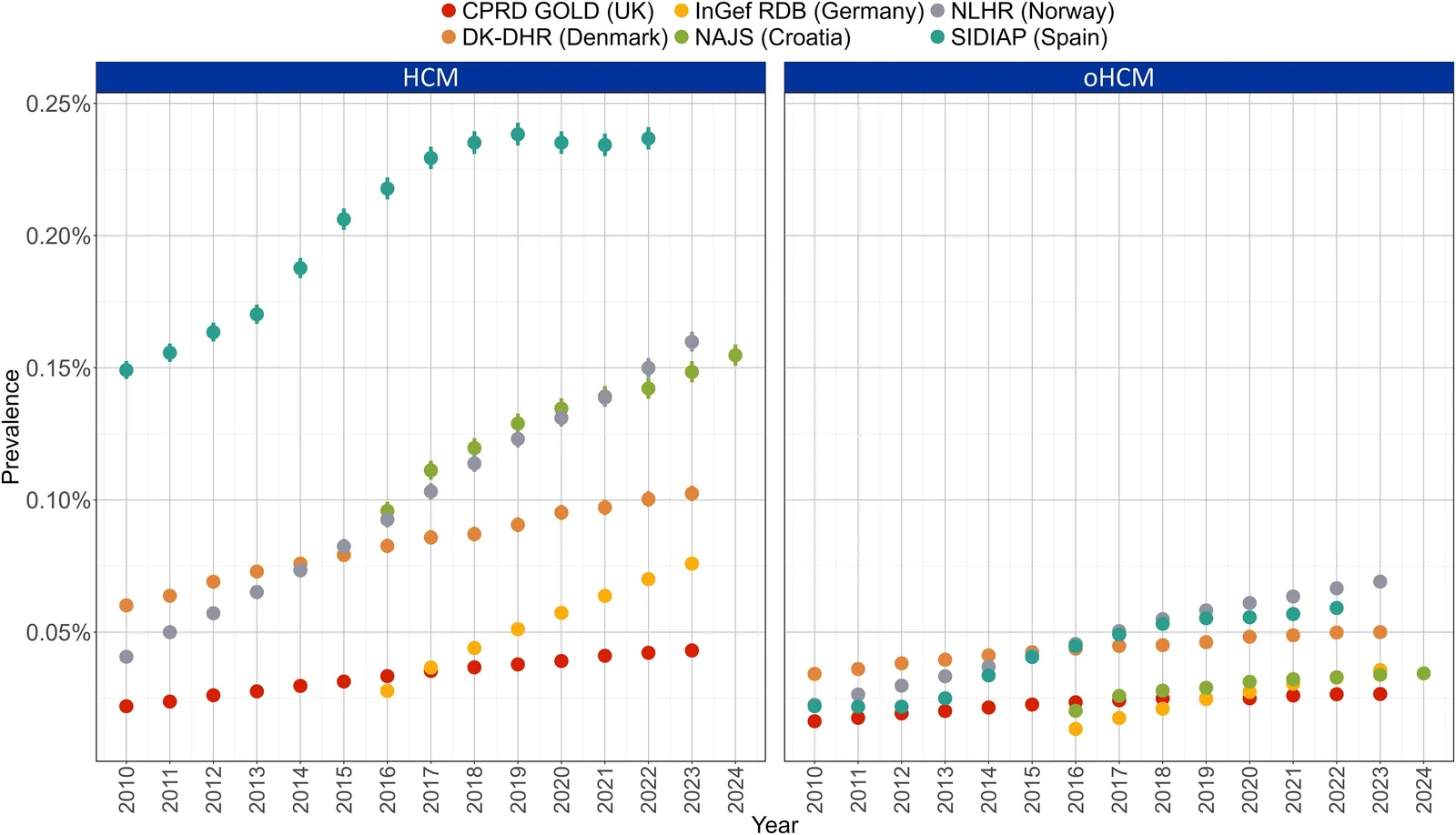
Annual period prevalence and 95% CI of clinically recognized HCM and oHCM cohorts by study database. All point estimates, as well as 95%CI, can be found in the interactive web application and in Supplementary Table S2. Notice that the prevalence estimate for 2015 in NAJS (the earliest available estimate) was excluded, as it was likely inflated, possibly due to all HCM or oHCM diagnoses before 2014 being recorded as starting in 2014 or later

First recorded clinically recognized HCM prevalence generally increased with age (interactive web application) in all databases, peaking at the oldest age groups, and was higher in males than in females. However, sex differences were less pronounced among older adults, particularly in the 80+ age group, where females had similar or slightly higher prevalence than males (except for SIDIAP). For first recorded clinically recognized oHCM, while prevalence remained higher in males than females, sex differences were smaller compared to the clinically recognized HCM cohort.

### Patient-level characterization

#### Comorbidities

The most frequently recorded comorbidities among individuals with clinically recognized HCM at any time prior the index date were essential hypertension (ranging from 31% in CPRD GOLD to 82% in NAJS), cardiac arrhythmia (16% in CPRD GOLD to 42% in SIDIAP), ischaemic heart disease (14% in CPRD GOLD to 32% in NAJS), HF (4% in CPRD GOLD to 30% in SIDIAP), and AF (5% in DK-DHR to 24% in SIDIAP) (*Table 1*). DK-DHR recorded low hypertension (<2%).

In the three months preceding the index date, cardiac arrhythmia (3% in CPRD GOLD to 29% in NAJS), ischaemic heart disease (2% in CPRD GOLD to 19% in NAJS), and HF (1% in CPRD GOLD to 15% in NLHR) remained the most common recorded conditions, with similar patterns three months after the index date (*Figure 2A*). NAJS consistently showed a higher proportion of comorbidities compared to other databases.

**Figure 2:**
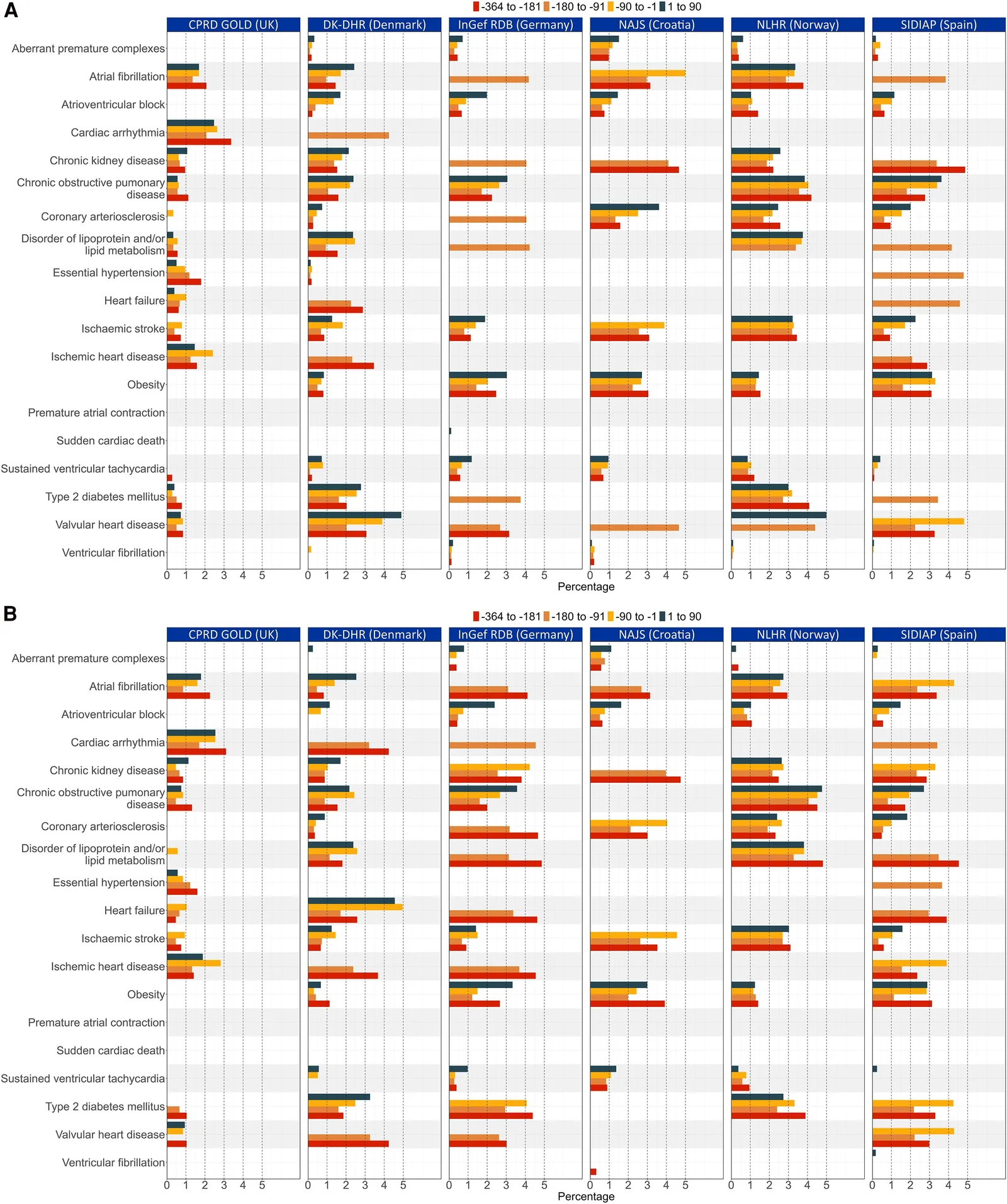
Frequency (%) of comorbidities by study database and time window (in days). In the panels the frequency of the comorbidities is shown for the clinically recognized (A) HCM cohort and (B) oHCM cohort. All time windows and exact frequencies can be found in the interactive web application

Recorded comorbidities in the clinically recognized oHCM cohort were similar to those observed in the HCM cohort (*Figure 2B*). Essential hypertension, disorder of lipoprotein and/or lipid metabolism, cardiac arrhythmia, and ischaemic heart disease were among the most frequently recorded comorbidities at any time prior to the index date.

Aberrant premature complexes, atrioventricular block, premature atrial contraction, sudden cardiac death, sustained ventricular tachycardia, and ventricular fibrillation had very low prevalence across all databases and in both HCM forms (Supplementary Figure S4).

#### Diagnostic measurements

Echocardiography was the most commonly recorded diagnostic measurement in the three months prior to the index date, being the highest in InGef RDB (21%), followed by NAJS (13%), SIDIAP (6%), and CPRD GOLD (2%) (*Figure 3A*). Cardiac catheterization was most frequent in InGef (34%), but was under 5% in other databases (NAJS, CPRD GOLD) and was not recorded in SIDIAP. Cardiac Magnetic Resonance Image (MRI) and left ventriculogram were also mainly recorded in InGef RDB (12% and 11%, respectively). Electrocardiography was most commonly recorded in NAJS (5%). Exercise test and endomyocardial biopsy were only recorded in CPRD GOLD and InGef RDB at low frequencies (<5%). Left ventricular ejection fraction (LVEF) was only recorded in SIDIAP (2%). No records existed for LVOT, genetic testing, or heart wall thickness.

**Figure 3:**
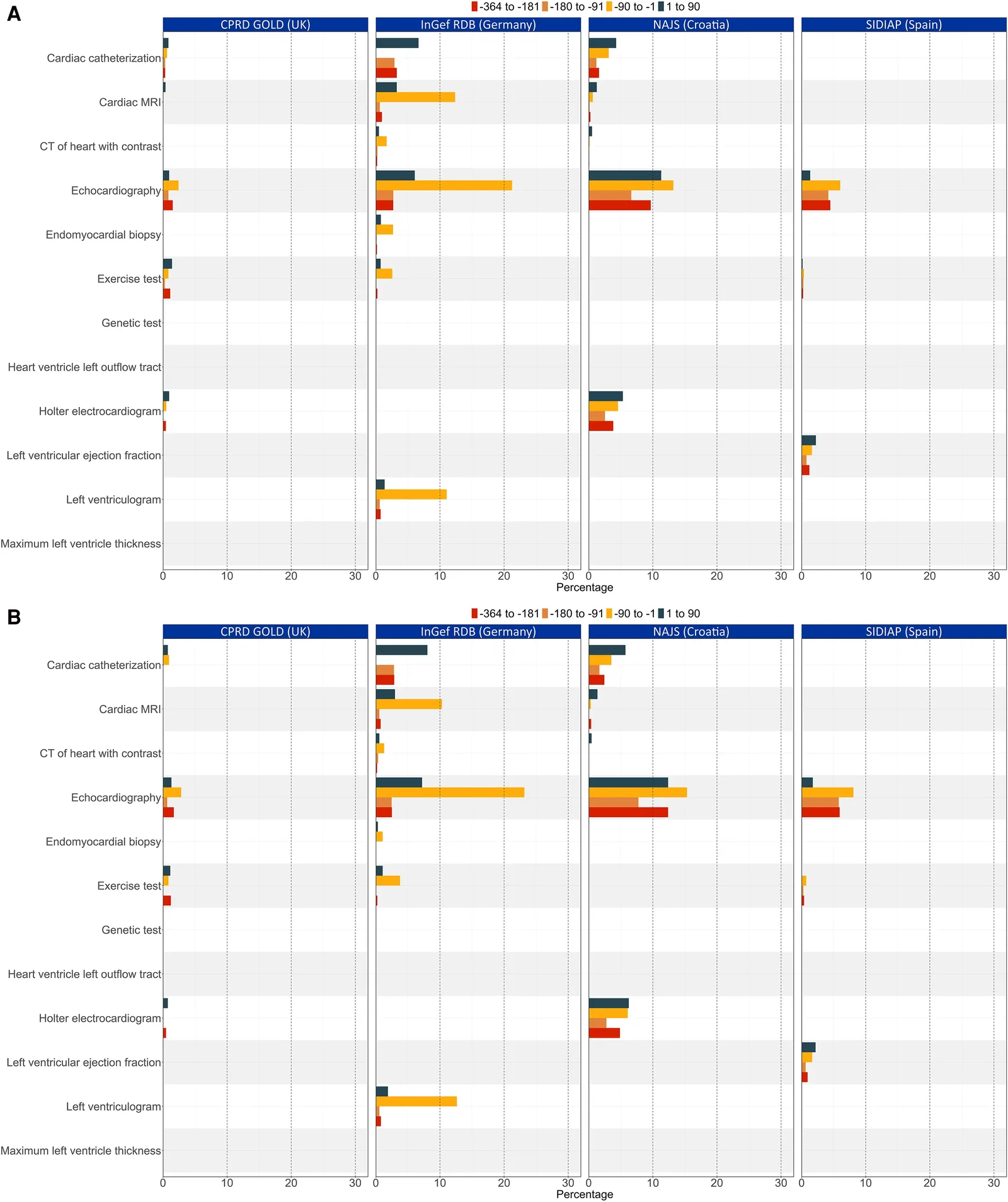
Frequency (%) of diagnostic measurements by study database and time windows (in days). In panels, the frequency of the diagnostic measurements is shown for the clinically recognized (A) HCM cohort and (B) oHCM cohort. All time windows and exact frequencies can be found in the interactive web application. Notice that DK-DHR and NLHR did not have data on measurements

Measurement patterns recorded in the clinically recognized oHCM cohort closely mirrored those in HCM (*Figure 3B*).

#### Medications

In the clinically recognized HCM cohort, beta-blockers were the most commonly recorded medication, ranging from 28% to 63% (NLHR—InGef RDB) at any time before the index date (interactive web application) and from 21% to 48% (DK-DHR—SIDIAP) in the three months prior (*Figure 4A*). Diuretics were also frequently recorded, with prevalence ranging from 16% to 60% (NLHR—SIDIAP) before the index date and 12% to 43% (NLHR—SIDIAP) in the three months prior. Angiotensin-Converting Enzyme (ACE) inhibitors ranged from 11% to 56% (InGef RDB—SIDIAP) before the index date and 9% to 29% (NLHR—SIDIAP) in the three months prior. In NLHR, after beta-blockers, angiotensin II receptor blockers and platelet aggregation inhibitors (excluding heparin) were also commonly recorded any time before the index date (21% and 19%, respectively), with similar trends three months prior (19% and 15%, respectively). These medications remained the most frequently recorded at and after the index date. Vitamin K antagonists were notably more common in NAJS and SIDIAP, with prescription records of over 10% both before and after the index date.

**Figure 4:**
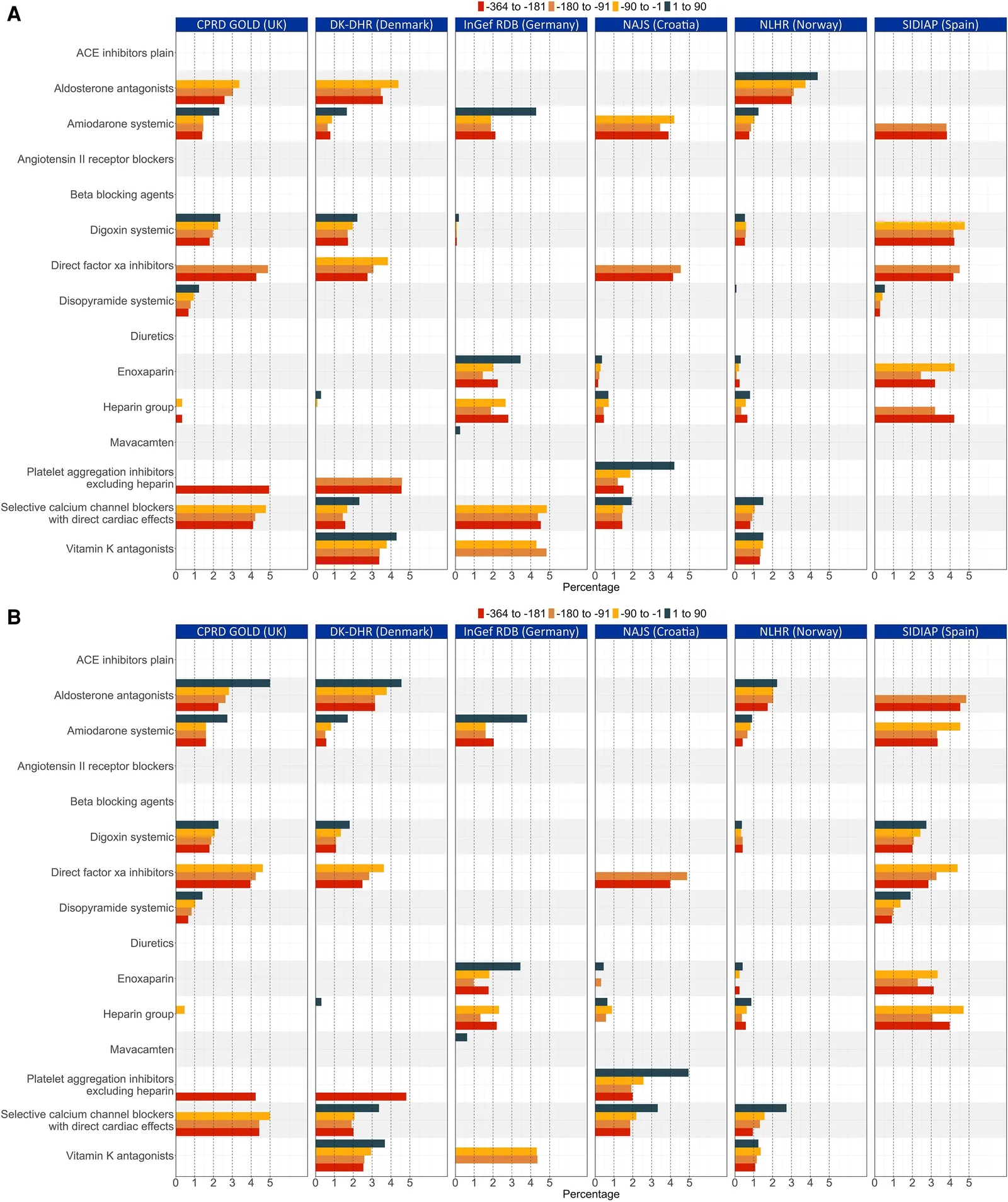
Frequency (%) of medications by study database and time window (in days). In panels, the frequency of the recorded medications is shown for the clinically recognized (A) HCM cohort and (B) oHCM cohort. All time windows and exact frequencies can be found in the interactive web application

Medications recorded in the clinically recognized oHCM cohort were similar to those observed in the overall HCM cohort. Three months post-index date, beta-blocker agents, ACE inhibitors, and diuretics remained the most frequently recorded medications (*Figure 4B*). However, selective calcium channel blockers were more commonly prescribed than in the clinically recognized HCM cohort (NAJS, SIDIAP), especially after the index date. Mavacamten was also more frequent in the clinically recognized oHCM cohort (InGef RDB), with prevalence peaking at 1.6% five or more years after the index date.

Enoxaparin, heparin, and disopyramide (systemic) were recorded at very low frequencies across all databases and in both HCM forms (Supplementary Figure S5).

#### Treatment procedures

In the clinically recognized HCM cohort, the most commonly recorded treatment procedures were pacemaker implantation, defibrillator implantation, and septal reduction therapy, with data available only on CPRD GOLD, InGef RDB, and NAJS (*Figure 5A*). InGef RDB reported the highest frequencies in the three months prior to index date (6%, 3%, and 2%, respectively), which declined after the first recorded diagnosis. Following the index date, defibrillator implantation frequencies peaked between 1 and 3 years in InGef RDB (2%), and after 5 or more years in CPRD GOLD (2%) and NAJS (1%) (interactive web application). Pacemaker implantation peaked 1–3 years post-index date in NAJS (6%) and InGef RDB (4%), and after 5 or more years in CPRD GOLD (3%). Heart valve transplantation was most frequent 5 or more-year post-index date (4% DK DHR, 3% InGef RDB, 2% NAJS and SIDIAP, 1% NLHR). Heart transplantation was rarely recorded.

**Figure 5:**
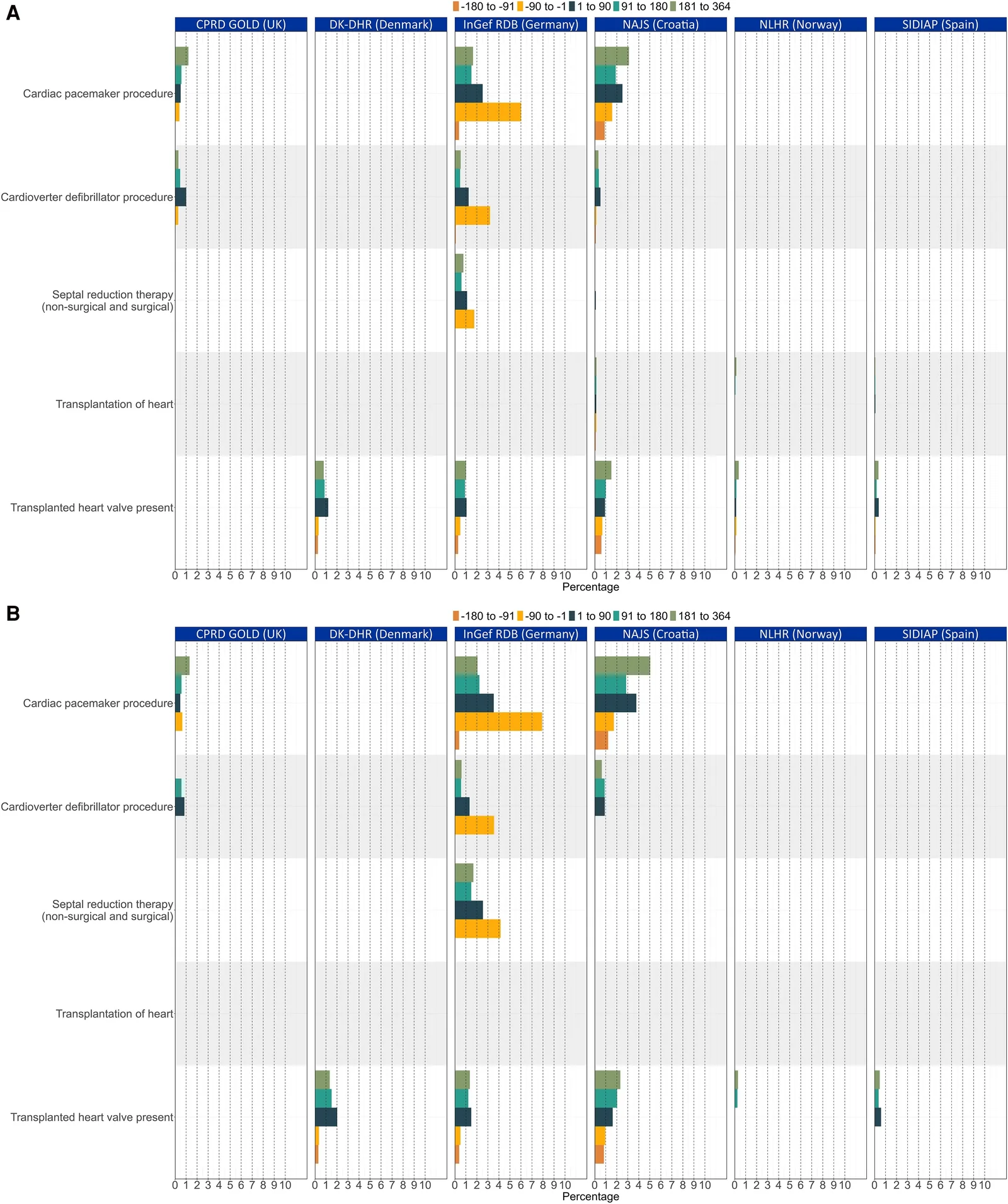
Frequency (%) of treatment procedures by study database and time window (in days). In the panels the frequency of the treatment procedures is shown for the clinically recognized (A) HCM cohort and (B) oHCM cohort. All time windows and exact frequencies can be found in the interactive web application

Procedures recorded in the clinically recognized oHCM cohort were largely similar to those observed in the HCM cohort, although septal reduction therapy was more common than in clinically recognized HCM (4% vs 2% in InGef RDB) 3 months prior to the index date (*Figure 5B*). Heart valve transplantation was also more frequent in patients with clinically recognized oHCM, doubling the proportion of individuals to 6% (DK-DHR) 5 or more years post-index date (interactive web application).

Supplementary Note S3 describes sex-stratified results. Briefly, females consistently showed a higher proportion of valvular heart disease, essential hypertension, and chronic kidney disease and were more often prescribed diuretics, while males showed a higher proportion of chronic obstructive pulmonary disease. Additionally, most of the comorbidities, measurements, and medications were first recorded more than one year before the index date in both clinically recognized HCM and oHCM cohorts, showing similar timing patterns between males and females (Supplementary Note S4).

## Discussion

### Key findings

Our study identified 40 277 individuals with clinically recognized HCM across six European databases, including 12 363 with oHCM. Females tended to be older than males at the time of first recorded diagnosis. Our study showed an increase in prevalence over time, with the most recent estimates ranging from 0.04% (CPRD GOLD, UK) to 0.24% (SIDIAP, Spain). Prevalence generally increased with age and was generally higher in males; however, older females (aged 80+ years) had similar or even higher prevalence than males in most databases, particularly in the oHCM cohorts.

Cardiovascular conditions were highly prevalent among individuals with clinically recognized HCM and oHCM, with essential hypertension, cardiac arrhythmia (including AF), ischaemic heart disease, and HF being the most commonly recorded. Beta-blockers, diuretics, and ACE inhibitors were among the most recorded medications. Notably, most comorbidities and medications were first recorded before the first documented diagnosis of HCM or oHCM, often more than a year earlier.

### Results in context

Since the first study published in 1995 (CARDIA),^26^ which estimated HCM prevalence at approximately 1 in 500 (0.2%) in the US based on imaging-identified left ventricular hypertrophy, several studies have used different methodologies to assess the frequency of HCM in the general population.^27^ While claims and electronic health records (EHR) data provide insights into clinical recognition, and genomic data help to identify at-risk populations, only imaging can establish a clinical diagnosis of HCM with certainty.^27^ In this study, using claims, registries, and EHR data from six European countries, prevalence estimates were highly variable. The lowest prevalence was observed in CPRD GOLD (primary care data from the UK), showing similar estimates to previous studies using EHR, such as a German study estimating 0.07% in 2015^28^ and studies from the UK and Sweden reporting 0.04%.^29,30^ In contrast, the higher prevalence found in SIDIAP (Catalan primary care data linked to hospital discharge data) more closely aligns with imaging- and genetic-based studies, with prevalences ranging from 0.11 in the UK to 1.4% in the USA.^31,32^ Notice that the German study,^28^ despite using the same database (InGef RDB), reported higher prevalences than detected in our study. This may likely be because at the time of our study, InGef RDB only included hospital diagnoses but not outpatient diagnoses, as the latter were not mapped to the OMOP common data model.

The increasing prevalence of clinically recognized HCM observed over time aligns with findings from other studies,^32,33^ and likely reflects improvements in diagnostic capabilities, clinicians’ awareness, and disease management strategies to potentially increase survival among individuals with HCM. In particular, the growing use of cardiac MRI, may have also contributed to a higher detection rate of HCM.^34^ However, we could not associate the increase in prevalence with advancement in imaging performance, as MRI data was only available for InGef RDB. Demographic factors, particularly population ageing, and healthcare system and data-related factors, including changes in healthcare utilization, reimbursement incentives, coding practices, and database maturation, may have also contributed to the observed increase in prevalence.

Sex and age distribution of clinically recognized HCM were consistent with those found in previous studies.^12,28,35–37^ Clinically recognized HCM prevalence increased with age, with most individuals diagnosed in their 60 s. Overall, males had a higher prevalence of clinically recognized HCM compared to females; however, older females had similar or even higher prevalence than their male counterparts. For clinically recognized oHCM, sex differences in prevalence were smaller, with females even outnumbering men in DK-DHR and SIDIAP. Additionally, females were generally older at the time of the first recorded HCM diagnosis and had higher recorded HF and valvular disease prevalence in some databases, as reported previously.^12,38,39^ Although the present study could not determine the reasons underlying these sex differences, several hypotheses may be considered. Later first recorded diagnosis in females may reflect differences in symptom onset, symptom recognition, or referral patterns. It may also suggest delayed detection, potentially resulting in diagnosis at a more advanced or obstructive stage. Alternatively, females may experience a higher rate of progression from non-obstructive to obstructive HCM. Differences in survival could also contribute to the higher proportion of women observed in older age groups, although existing evidence has reported worse overall survival among females with HCM.^40^ Together, these hypotheses are consistent with previously described sex-related biases in HCM, potentially leading to delayed diagnoses and underutilization of specialized treatment for females^41,42^; however, they could not be disentangled in this study, and further research is needed to clarify the underlying mechanisms. In addition, differences in healthcare utilization, coding practices, and the underlying age structure of the participating databases may also have contributed to the observed sex-specific patterns.

The most commonly recorded comorbidities among individuals with clinically recognized HCM (essential hypertension, cardiac arrhythmias, ischaemic heart disease, and HF) and medications (beta-blockers, diuretics, and ACE inhibitors) were consistent with those observed in other studies,^12,28,30,43^ and in line with clinical guidelines.^44^ Most of these were consistently first recorded over a year before the first recorded HCM diagnosis, suggesting that first recorded diagnosis of clinically recognized HCM often occurred after a prolonged period of experiencing symptoms or comorbidities, although these findings may also be influenced by underlying comorbidity burden, diagnostic overlap, and database-specific recording practices. Two UK studies have previously reported that HCM-related comorbidities and treatments were already recorded within the year preceding diagnosis^29,45^: Magnusson *et al.* reported stable angina (24%), AF (16%), and HF (13%) as the most commonly recorded comorbidities, with almost 54% of them receiving blood pressure-lowering medication, 18% of patients on statins, 9% receiving anticoagulants, and 9% treated with amiodarone; Brownrigg *et al.* reported hypertension (54%), coronary artery disease (30%), AF (26%), and valvular heart disease (17%) as the most common comorbidities, with 5% of patients having an implantable cardioverter defibrillator and 7% a pacemaker.

Essential hypertension, which we found to be the most common recorded comorbidity and was highly prevalent before the first recorded HCM or oHCM diagnosis, has previously been described in patients with HCM, with proportions ranging from 31% to 83%.^28,43,46,47^ Notably, DK-DHR showed very low rates of essential hypertension (<2% of individuals with clinically recognized HCM), likely reflecting underreporting of this condition (see the full study report for further details^25^). Traditionally, the presence of hypertension has led to reluctance in accepting a concurrent diagnosis of HCM, as hypertension is known to also cause left ventricular hypertrophy. As a result, hypertension has been associated with a delayed diagnosis of HCM,^48^ and some studies estimating the prevalence of HCM have even excluded individuals with essential hypertension from their HCM cohorts due to its potential confounding role.^27^Given that our study relied on recorded diagnosis of HCM or oHCM without an imaging, haemodynamic, or genetic test confirmation, the high prevalence of hypertension observed in our cohorts may partly reflect diagnostic misclassification between hypertensive heart disease and HCM. However, excluding individuals with hypertension would overlook its significant role as a common comorbidity in HCM. From a clinical perspective, in patients with oHCM, the coexistence of hypertension may further exacerbate left ventricular pressures, potentially worsening symptoms and increasing the risk of adverse outcomes. Therefore, a better understanding of the coexistence of these conditions is needed. Similarly, other common cardiovascular conditions, including valvular heart disease, ischaemic heart disease, and heart failure, may coexist with HCM while also contributing to diagnostic uncertainty and potential misclassification of HCM in routinely collected healthcare data.

### Strengths and limitations

To the best of our knowledge, this is the largest epidemiological study to simultaneously report on the prevalence and characteristics of patients with a recorded diagnosis of clinically recognized HCM across six European databases, covering a wide range of healthcare settings (hospital inpatient and outpatient care, secondary care outpatient settings, and primary care). Additionally, the harmonization of the data to a common data model allowed us to standardize phenotype definitions across the different databases.

However, our study had some limitations that must be considered. First, the analysis was restricted to individuals with diagnoses recorded from 2010 onwards, which ensured a consistent recording of diagnoses across databases and a more reliable identification of the first recorded diagnosis. However, longstanding cases may not be fully captured. As a result, our estimates reflect clinically recognized cases within the database and may underestimate the true population prevalence. This may also contribute to lower estimates in the earlier study years and to the apparent increase in prevalence over time, which may partly reflect the accumulation of diagnosed cases within the observable data. Second, although we included a wide range of routinely collected data sources (EHRs, claims, and registries), the four databases containing measurement records showed very low (or inexistent) recorded values of echocardiographic parameters, LVOT gradients, wall thickness, and genetic testing. This likely reflects incomplete capture of these data within routinely collected healthcare databases rather than a true absence of diagnostic evaluation, which restricted validation of recorded HCM diagnoses and impairs reliable assessment of disease severity and subtype, including differentiation between obstructive and non-obstructive forms or other causes of left ventricular hypertrophy. Third, we found high heterogeneity in clinically recognized HCM and oHCM prevalence across databases, likely due to variation in demographic characteristics, diagnostic and treatment practices, health care setting, the type of data captured, and coding practices. While age-standardization accounted for differences in age structure, other potential confounding factors could not be adjusted for. Accordingly, differences in the recorded proportion of oHCM between databases should not be interpreted as evidence of true epidemiological differences in obstructive disease. Similarly, there was a high heterogeneity in recorded comorbidity frequency, likely attributed also to demographic and healthcare coverage differences across databases. For example, NAJS, which had an overall higher frequency of recorded comorbidities over time, included older individuals compared to CPRD GOLD, where the populations were younger. Differences in recording practices may have also contributed to this variability. For instance, NAJS likely captures repeated entries of the same condition at each patient visit, whereas CPRD GOLD or DK-DHR may document comorbidities only once. Fourth, the recorded use of treatments should be interpreted in the context of the available data, as the underlying indication for prescribing could not be assessed. Consequently, medications such as beta-blockers, diuretics, and ACE inhibitors may reflect treatment for coexisting comorbidities, rather than HCM-specific management alone. Lastly, the COVID-19 pandemic (2020–2022) introduced additional challenges by altering healthcare utilization patterns, which may have impacted the identification and reporting of clinically recognized HCM and oHCM. Interestingly, the increasing prevalence trend continued through 2020–2021 in most databases. The only exception was SIDIAP, where a slight decrease in clinically recognized HCM prevalence was observed during this period, which may reflect local disruptions in healthcare access or coding practices during the pandemic.

## Conclusion

Our study shows a consistent annual increase in the prevalence of clinically recognized HCM and oHCM across six European healthcare settings, with cardiovascular comorbidities and their associated treatments frequently recorded more than one year before the first recorded HCM diagnosis. The observed increase in prevalence is likely multifactorial and may reflect demographic changes, improvements in disease recognition, evolving clinical practice, database-specific factors, and potentially changes in the underlying disease burden. These findings highlight the importance of maintaining diagnostic awareness among clinicians to facilitate timely identification of the disease and may help inform the development and evaluation of future screening and management strategies, including the introduction of emerging treatments such as *mavacamten*.

## Supplementary Material

xvag218_Supplementary_Data

## Declarations

### Data Availability Data Access

Analyses were run separately for each database using federated network analyses. The DARWIN EU® Coordination Centre executed the analytics on a subset of data sources or a simulated set of patients, with quality control checks implemented. Data partners locally executed the analysis using OMOP CDM in RStudio, reviewed and approved the by-default aggregated results before returning them to the DARWIN EU® Coordination Centre. All results were locked and timestamped to ensure reproducibility and transparency.

### Data Sharing

All databases were previously mapped to the OMOP-CDM. This enabled the use of standardized analytics and tools across the network since the structure of the data and the terminology system was harmonized. The analytic code for this study was written in R. Each data partner executed the study code against their database containing patient-level data and then returned the results set, which only contained aggregated data. The results from each of the contributing data sites had been combined in tables and figures for the study report. Access to patient-level data is specific to each database and must be requested individually from each data source. Aggregated data is fully available in the interactive web application of the study: https://data.darwin-eu.org/HCM_Characterisation_Manuscript/. The code of the study is fully available in GitHub: https://github.com/darwin-eu-studies/EUPAS1000000430.

### Pre-registered Clinical Trial Number

None supplied.
