# Supplementary material for: Characterizing clinically recognized hypertrophic cardiomyopathy in six European countries using real-world data
Source: ESC Heart Fail. 2026 Aug 20;13(5):xvag218. doi: 10.1093/eschf/xvag218 (PMC13639481; doi:10.1093/eschf/xvag218)
Supplement: Supplementary file 1 [file xvag218_supplementary_data.docx]

# **Supplementary Note 1.** SNOMED code lists (vocabulary version 5.0) used to define clinically recognised hypertrophic cardiomyopathy (HCM), obstructive HCM (oHCM), predefined comorbidities, diagnostic measurements, medications, treatment procedures and other covariates.

Main conditions of interest

| **Name** | **Concept ID** |
| --- | --- |
| **Hypertrophic cardiomyopathy without obstruction (HCM)** | |
| Hypertrophic cardiomyopathy without obstruction | 4108236 |
| Hypertrophic cardiomyopathy | 4124693 |
| Hypertrophic obstructive cardiomyopathy | 316428 |
| Hypertrophic cardiomyopathy with genetic marker | 43020658 |
| Primary idiopathic hypertrophic cardiomyopathy | 4270625 |
| Primary hypertrophic cardiomyopathy | 44784145 |
| Primary familial hypertrophic cardiomyopathy | 4222765 |
| **Hypertrophic cardiomyopathy with obstruction (oHCM)** | |
| Hypertrophic obstructive cardiomyopathy | 316428 |

Covariates

To study the annual prevalence, age and sex (female/male) at first recorded HCM or oHCM diagnosis were described. The following age grouping was used to stratify the prevalence: 18-29, 30-39, 40-49, 50-59, 60-69, 70-79, 80 and over.

For the patient-level characterisation, predefined comorbidities, measurements and treatments were assessed in different time windows before, at the time of, and after the first recorded HCM or oHCM diagnosis. Windows included are: any time prior to index date (-Inf, -1], >5 years (-Inf, -1825], 5 to 3 years [-1824, -1095], <3 to 1 year [-1094, -365], -364 to -181 days [-364, -181], -180 to -91 days [-180, -91], -90 to -1 [-90, -1] days before the index date, at index date [0,0], and during the periods 1 to 90 days [1,90], 91 to 180 days [91,180], 181 to 364 days [181,364], 1 to <3 years [365, 1094], 3 to 5 years [1095, 1824], >5 years after [1825, Inf). For medications, number of persons initiating or continuing the medication in the respective time windows are reported.

Predefined comorbidities

Note that all descendants of these concepts were also included. The following concepts were excluded:

- For heart failure: Postpartum cardiomyopathy (312383), Kyphoscoliotic heart disease (317000), Congenital cardiac failure (4071869), Neonatal cardiac failure (4172864), Rheumatic left ventricular failure (4184497), and Cardiac asthma (4215802)
- For ischaemic heart disease: Old myocardial infarction (314666)
- For obesity: Hypertrophy of fat pad of knee (380500), Childhood obesity (40481140), Drug-induced obesity (4097996), Alstrom syndrome (4270189)
- Type 2 diabetes: Type 2 diabetes in remission (45766052)

| **Comorbidities** | **Concept name** | **Concept ID** |
| --- | --- | --- |
| Aberrant premature complexes | Aberrant premature complexes | 4091901 |
|  | Ventricular premature complex | 4089462 |
|  | Atrial premature complex | 4115173 |
| Atrial fibrillation | Atrial fibrillation | 313217 |
|  | Paroxysmal atrial fibrillation | 4154290 |
|  | Persistent atrial fibrillation | 4232697 |
|  | Chronic atrial fibrillation | 4141360 |
|  | Atrial fibrillation and flutter | 4108832 |
|  | Permanent atrial fibrillation | 4232691 |
| Atrioventricular block | Atrioventricular block | 316135 |
|  | Complete atrioventricular block | 320744 |
|  | First degree atrioventricular block | 314379 |
|  | Second degree atrioventricular block | 318448 |
| Cardiac arrhythmia | Cardiac arrhythmia | 44784217 |
|  | Atrial arrhythmia | 4068155 |
|  | Supraventricular tachycardia | 4275423 |
|  | Typical atrial flutter | 36714994 |
|  | Left bundle branch block | 316998 |
|  | Right bundle branch block | 314059 |
|  | Left anterior fascicular block | 4295336 |
|  | Sick sinus syndrome | 4261842 |
|  | Re-entry ventricular arrhythmia | 4111552 |
|  | Bifascicular block | 4250169 |
|  | Arrhythmia during surgery | 37108582 |
|  | Ventricular tachyarrhythmia | 40622721 |
|  | Sudden arrhythmic death syndrome | 42536727 |
|  | Supraventricular bradyarrhythmia | 42538755 |
|  | Cardiac arrhythmia | 44784217 |
|  | Cardiac arrhythmia in mother complicating childbirth | 45757098 |
|  | Tachyarrhythmia | 315643 |
|  | EKG: ventricular arrhythmia | 4064453 |
|  | EKG: supraventricular arrhythmia | 4065287 |
|  | Atrial arrhythmia | 4068155 |
|  | Marked sinus arrhythmia | 4088347 |
|  | Re-entry ventricular arrhythmia | 4111552 |
|  | ECG: sinus arrhythmia | 4141820 |
|  | Ventricular arrhythmia | 4185572 |
|  | Bradyarrhythmia | 4228448 |
|  | AV node arrhythmia | 4228836 |
|  | Supraventricular arrhythmia | 4248028 |
|  | Normal sinus arrhythmia | 4313053 |
|  | Premature beats | 316429 |
|  | Nodal rhythm disorder | 4217221 |
|  | Neonatal dysrhythmia | 4173170 |
|  | Left bundle branch hemiblock | 313209 |
|  | Atrial fibrillation | 313217 |
|  | Anomalous atrioventricular excitation | 313224 |
|  | Mobitz type II atrioventricular block | 313780 |
|  | Bundle branch block | 313791 |
|  | Paroxysmal tachycardia | 313792 |
|  | Right bundle branch block | 314059 |
|  | First degree atrioventricular block | 314379 |
|  | Long QT syndrome | 314664 |
|  | Atrial flutter | 314665 |
|  | Atrioventricular block | 316135 |
|  | Right bundle branch block AND left anterior fascicular block | 316432 |
|  | Left bundle branch block | 316998 |
|  | Conduction disorder of the heart | 316999 |
|  | Sinus node dysfunction | 317302 |
|  | Paroxysmal supraventricular tachycardia | 317893 |
|  | Second degree atrioventricular block | 318448 |
|  | Heart block | 320425 |
|  | Complete atrioventricular block | 320744 |
|  | Trifascicular block | 321315 |
|  | Bilateral bundle branch block | 321587 |
|  | Right bundle branch block AND left posterior fascicular block | 321590 |
|  | Paroxysmal ventricular tachycardia | 437579 |
|  | Ventricular fibrillation | 437894 |
|  | Supraventricular premature beats | 441872 |
|  | Neonatal bradycardia | 443522 |
|  | Neonatal tachycardia | 443523 |
|  | Ventricular bigeminy | 4008580 |
|  | Multifocal PVCs | 4023336 |
|  | Sinoatrial arrest with nodal/ventricular escape | 4028322 |
|  | Fusion beats | 4029303 |
|  | Right bundle branch block, posterior fascicular block AND incomplete left bundle branch block | 4032785 |
|  | Monofascicular block | 4034164 |
|  | AV junctional rhythm | 4038688 |
|  | Accelerated atrioventricular conduction | 4057008 |
|  | Ventricular premature beats | 4066289 |
|  | Atrioventricular tachycardia | 4081675 |
|  | Sinus arrest with junctional escape | 4088210 |
|  | Intermittent second degree atrioventricular block | 4088332 |
|  | Incomplete left bundle branch block | 4088336 |
|  | Complete right bundle branch block | 4088337 |
|  | Incomplete right bundle branch block | 4088338 |
|  | Slow ventricular response | 4088350 |
|  | Junctional premature complex | 4088351 |
|  | Sinus arrest with atrial escape | 4088352 |
|  | Minor intraventricular conduction defect | 4088496 |
|  | AV junctional (nodal) arrest | 4088502 |
|  | AV junctional (nodal) tachycardia | 4088503 |
|  | Atrial bigeminy | 4088504 |
|  | Ventricular trigeminy | 4088505 |
|  | Ventricular quadrigeminy | 4088506 |
|  | Ventricular escape complex | 4088507 |
|  | AV-junctional (nodal) bradycardia | 4088983 |
|  | Blocked premature atrial contraction | 4088984 |
|  | Multiple premature ventricular complexes | 4088985 |
|  | Atrial escape complex | 4088986 |
|  | Atrial parasystole | 4088987 |
|  | AV nodal re-entry tachycardia | 4089459 |
|  | Multiple atrial premature complexes | 4089460 |
|  | Run of atrial premature complexes | 4089461 |
|  | Ventricular premature complex | 4089462 |
|  | Multiple ventricular interpolated complexes | 4089463 |
|  | Paired ventricular premature complexes | 4089464 |
|  | Sinus arrest with ventricular escape | 4091446 |
|  | Junctional ectopic tachycardia | 4091899 |
|  | Aberrant premature complexes | 4091901 |
|  | Supraventricular bigeminy | 4091902 |
|  | Atrial trigeminy | 4091903 |
|  | Run of ventricular premature complexes | 4091904 |
|  | Ventricular interpolated complexes | 4092010 |
|  | Aberrantly conducted complex | 4092011 |
|  | Chronic ectopic atrial tachycardia | 4098133 |
|  | Unifocal PVCs | 4099778 |
|  | Asymptomatic sinoatrial node dysfunction | 4102252 |
|  | Vagal autonomic bradycardia | 4106715 |
|  | Paroxysmal atrioventricular tachycardia | 4108241 |
|  | Ventricular pre-excitation | 4108828 |
|  | Atrial paroxysmal tachycardia | 4108830 |
|  | Atrial fibrillation and flutter | 4108832 |
|  | Premature atrial contraction | 4109365 |
|  | Left main stem bundle branch block | 4111543 |
|  | Paroxysmal junctional tachycardia | 4111546 |
|  | Partial atrioventricular block | 4111570 |
|  | Paroxysmal nodal tachycardia | 4111698 |
|  | Ventricular fibrillation and flutter | 4111700 |
|  | Atrial premature complex | 4115173 |
|  | Controlled atrial fibrillation | 4117112 |
|  | Lone atrial fibrillation | 4119601 |
|  | Non-rheumatic atrial fibrillation | 4119602 |
|  | Postoperative complete heart block | 4119605 |
|  | Sinoatrial node tachycardia | 4120084 |
|  | Supraventricular tachycardia with functional bundle branch block | 4120085 |
|  | His bundle tachycardia | 4120086 |
|  | Unidirectional retrograde accessory pathway | 4120087 |
|  | Ectopic atrial tachycardia | 4121479 |
|  | Ectopic atrioventricular node tachycardia | 4121480 |
|  | Antidromic atrioventricular re-entrant tachycardia | 4121481 |
|  | Re-entrant atrial tachycardia | 4124696 |
|  | Incessant atrial tachycardia | 4124697 |
|  | Re-entrant atrioventricular node tachycardia | 4124698 |
|  | Re-entrant atrioventricular tachycardia | 4124699 |
|  | Orthodromic atrioventricular re-entrant tachycardia | 4124700 |
|  | Postoperative His bundle tachycardia | 4124701 |
|  | Permanent junctional reciprocating tachycardia | 4124702 |
|  | Postoperative sinus node dysfunction | 4124704 |
|  | Chronic atrial flutter | 4137382 |
|  | Right bundle branch block, anterior fascicular block AND posterior fascicular block | 4138545 |
|  | Right bundle branch block with left bundle branch block | 4138973 |
|  | Chronic atrial fibrillation | 4141360 |
|  | Ectopic beats | 4143042 |
|  | Paroxysmal atrial flutter | 4146580 |
|  | Lev's syndrome | 4153404 |
|  | Paroxysmal atrial fibrillation | 4154290 |
|  | Ectopic rhythm | 4164083 |
|  | Junctional escape beats | 4166380 |
|  | Intraventricular conduction defect | 4166844 |
|  | Severe sinus bradycardia | 4169261 |
|  | Idioventricular rhythm | 4171193 |
|  | Atrial tachycardia | 4171269 |
|  | Sinus bradycardia | 4171683 |
|  | Left atrial incisional tachycardia | 4171887 |
|  | Atrioventricular dissociation | 4175473 |
|  | Multifocal atrial tachycardia | 4176112 |
|  | Right bundle branch block AND incomplete left bundle branch block | 4184950 |
|  | Stokes-Adams syndrome | 4188347 |
|  | Paroxysmal atrial tachycardia with block | 4190306 |
|  | Nonparoxysmal AV nodal tachycardia | 4191222 |
|  | Rapid atrial fibrillation | 4199501 |
|  | Mobitz type I incomplete atrioventricular block | 4205137 |
|  | Sinus arrest | 4210313 |
|  | Right bundle branch block, anterior fascicular block AND incomplete posterior fascicular block | 4217860 |
|  | Ventricular escape rhythm | 4218242 |
|  | Junctional premature beats | 4218739 |
|  | Irregular tachycardia | 4221549 |
|  | Fibrillation | 4226399 |
|  | Permanent atrial fibrillation | 4232691 |
|  | Persistent atrial fibrillation | 4232697 |
|  | Ectopic atrial beats | 4236004 |
|  | Interpolated PVCs | 4243143 |
|  | Right bundle branch block, anterior fascicular block AND incomplete left bundle branch block | 4244693 |
|  | Ventricular parasystole | 4244893 |
|  | Accelerated idioventricular rhythm | 4247537 |
|  | Right branch block, incomplete anterior fascicular block AND incomplete posterior fascicular block | 4249027 |
|  | Bifascicular block | 4250169 |
|  | Tachycardia-bradycardia | 4254116 |
|  | Persistent sinus bradycardia | 4258998 |
|  | Sick sinus syndrome | 4261842 |
|  | Tic-tac rhythm | 4262389 |
|  | Complete left bundle branch block | 4267892 |
|  | Left posterior fascicular block | 4268046 |
|  | Multifocal premature beats | 4271464 |
|  | Supraventricular tachycardia | 4275423 |
|  | Sinoatrial block | 4277903 |
|  | Right bundle branch block, posterior fascicular block AND incomplete anterior fascicular block | 4280348 |
|  | Left anterior fascicular block | 4295336 |
|  | Anterior fascicular block, posterior fascicular block AND incomplete right bundle branch block | 4296729 |
|  | Incomplete atrioventricular block with atrioventricular response | 4298806 |
|  | Cardiac arrest due to pacemaker failure | 4301015 |
|  | Right atrial incisional tachycardia | 4302802 |
|  | Catecholaminergic polymorphic ventricular tachycardia | 4303238 |
|  | Sinoatrial nodal reentrant tachycardia | 4303256 |
|  | Diffuse intraventricular block | 4304839 |
|  | Atrioventricular conduction disorder | 4305210 |
|  | Idiojunctional tachycardia | 4320474 |
|  | Sustained ventricular fibrillation | 4325850 |
|  | Ventricular escape beat | 4327066 |
|  | Atypical atrial flutter | 36712986 |
|  | Typical atrial flutter | 36714994 |
|  | Chronic atrial and intestinal dysrhythmia | 36715042 |
|  | Idiopathic ventricular fibrillation not Brugada type | 37109917 |
|  | Acquired complete atrioventricular block | 37116420 |
|  | Acquired Brugada syndrome | 37312140 |
|  | Scar mediated macro re-entrant atrial tachycardia | 37312595 |
|  | Idiopathic neonatal atrial flutter | 37395937 |
|  | Long QT syndrome caused by drug | 37396235 |
|  | Acquired long QT syndrome | 40479264 |
|  | Symptomatic sinus bradycardia | 40480216 |
|  | Heart block due to drug | 40481891 |
|  | Bundle branch reentrant ventricular tachycardia | 40484036 |
|  | Macro re-entrant atrial tachycardia | 42536724 |
|  | Cavotricuspid isthmus dependent macroreentry tachycardia | 42536725 |
|  | Atrioventricular reciprocating tachycardia | 42536726 |
|  | Non-cavotricuspid isthmus dependent atrial tachycardia | 42539038 |
|  | Preexcited atrial fibrillation | 42539346 |
|  | Atrial standstill | 42872924 |
|  | High degree second degree atrioventricular block | 43020494 |
|  | Postoperative atrioventricular block | 43020495 |
|  | Atrioventricular block due to endocarditis | 43020929 |
|  | Atrial fibrillation with rapid ventricular response | 44782442 |
|  | Progressive familial heart block, type II | 44782643 |
|  | Nonsustained paroxysmal ventricular tachycardia | 44782707 |
|  | Nonsustained paroxysmal supraventricular tachycardia | 44782789 |
|  | Non-specific intraventricular conduction delay | 44784220 |
|  | Short QT syndrome | 44784236 |
|  | Longstanding persistent atrial fibrillation | 45768480 |
|  | Complete atrioventricular block as complication of atrioventricular nodal ablation | 46269694 |
|  | Mahaim fiber tachycardia | 46272503 |
|  | HHS - holiday heart syndrome | 46284985 |
|  | ECG: atrial fibrillation | 4064452 |
|  | Ventricular tachycardia | 4103295 |
|  | EKG: left bundle branch block | 4064614 |
|  | EKG: atrial flutter | 4065288 |
|  | EKG: right bundle branch block | 4064460 |
|  | EKG: heart block | 4064457 |
|  | Electrocardiogram: paroxysmal atrial tachycardia | 4064869 |
|  | Mobitz type I second degree atrioventricular block on electrocardiogram | 4064459 |
|  | Bifascicular block on electrocardiogram | 40483798 |
|  | Trifascicular block on electrocardiogram | 40482938 |
|  | EKG: Mobitz type II atrioventricular block | 4138921 |
|  | ECG: ventricular ectopics | 4064612 |
|  | EKG: atrial ectopics | 4065285 |
|  | Left anterior fascicular block on electrocardiogram | 40482086 |
|  | ECG: partial atrioventricular block - long PR | 4064873 |
|  | ECG: ectopic beats | 4064867 |
|  | Ventricular flutter | 433225 |
|  | EKG: ventricular fibrillation | 4064455 |
|  | Right bundle branch block and left anterior fascicular block on electrocardiogram | 40482887 |
|  | EKG: complete atrioventricular block | 4064874 |
|  | ECG: partial atrioventricular block - 2:1 | 4065290 |
| Chronic kidney disease | Chronic kidney disease | 46271022 |
|  | Chronic kidney disease stage 3 | 443597 |
|  | Chronic kidney disease stage 2 | 443601 |
|  | Chronic kidney disease stage 4 | 443612 |
|  | Chronic kidney disease stage 5 | 443611 |
|  | Chronic renal failure | 198185 |
|  | Chronic kidney disease due to hypertension | 44782429 |
|  | Hypertensive heart and chronic kidney disease | 44784621 |
|  | Chronic kidney disease due to type 2 diabetes mellitus | 43531578 |
|  | Anemia in chronic kidney disease | 45768812 |
|  | Chronic kidney disease stage 3B | 45763855 |
|  | Chronic kidney disease stage 3A | 45763854 |
| Chronic obstructive pulmonary disease | Chronic obstructive pulmonary disease | 255573 |
|  | Chronic obstructive pulmonary disease with acute lower respiratory infection | 4110056 |
|  | Moderate chronic obstructive pulmonary disease | 4193588 |
|  | Acute exacerbation of chronic obstructive pulmonary disease | 257004 |
|  | Acute exacerbation of chronic obstructive airways disease | 257004 |
| Coronary arteriosclerosis | Coronary arteriosclerosis | 317576 |
| Disorder of lipoprotein AND OR lipid metabolism | Disorder of lipoprotein AND/OR lipid metabolism | 4170226 |
|  | Disorder of lipid metabolism | 437530 |
|  | Pure hypercholesterolemia | 437827 |
|  | Hyperlipidemia | 432867 |
|  | Mixed hyperlipidemia | 438720 |
|  | Pure hyperglyceridemia | 440360 |
|  | Hyperglycemia due to type 2 diabetes mellitus | 37016349 |
| Essential hypertension | Essential hypertension | 320128 |
|  | Hypertensive disorder | 316866 |
| Heart failure | Heart failure | 316139 |
|  | Hypertensive heart disease with congestive heart failure | 314378 |
|  | Congestive heart failure | 319835 |
|  | Hypertensive heart and renal disease with both (congestive) heart failure and renal failure | 439694 |
|  | Diastolic heart failure | 443587 |
|  | Hypertensive heart failure | 444101 |
|  | Left heart failure | 439846 |
|  | Hypertensive heart and renal disease with (congestive) heart failure | 439696 |
|  | Chronic diastolic heart failure | 40479576 |
|  | Acute on chronic systolic heart failure | 40480602 |
|  | Right ventricular failure | 4273632 |
| Ischaemic stroke | Ischaemic stroke | 35826270 |
|  | Cerebral infarction | 443454 |
|  | Transient cerebral ischemia | 373503 |
|  | Cerebral ischemia | 374384 |
|  | Cerebrovascular accident | 381316 |
| Ischemic heart disease | Ischemic heart disease | 4185932 |
|  | Chronic ischemic heart disease | 315286 |
|  | Left heart failure | 439846 |
|  | Acute non-ST segment elevation myocardial infarction | 4270024 |
|  | Acute myocardial infarction | 312327 |
|  | Generalized ischemic myocardial dysfunction | 4110961 |
|  | Acute coronary syndrome | 4215140 |
|  | Angina pectoris | 321318 |
|  | Biventricular congestive heart failure | 4242669 |
| Obesity | Obesity | 433736 |
|  | Simple obesity | 4217557 |
|  | Obesity | 433736 |
|  | Extreme obesity with alveolar hypoventilation | 4100857 |
| Premature atrial contraction | Premature atrial contraction | 4109365 |
| Cardiac arrhythmia | Sick sinus syndrome | 4261842 |
| Sudden cardiac death | Sudden cardiac death | 4317150 |
| Sustained ventricular tachycardia | Sustained ventricular tachycardia | 4139206 |
|  | Ventricular tachycardia | 4103295 |
| Type 2 diabetis mellitus | Type 2 diabetis mellitus | 201826 |
|  | Type 2 diabetes mellitus without complication | 4193704 |
|  | Disorder due to type 2 diabetes mellitus | 443732 |
|  | Renal disorder due to type 2 diabetes mellitus | 443731 |
|  | Polyneuropathy due to diabetes mellitus | 376112 |
|  | Disorder of nervous system due to type 2 diabetes mellitus | 376065 |
|  | Hyperglycemia due to type 2 diabetes mellitus | 37016349 |
|  | Chronic kidney disease due to type 2 diabetes mellitus | 43531578 |
|  | Disorder of eye due to type 2 diabetes mellitus | 443733 |
|  | Hyperosmolar coma due to type 2 diabetes mellitus | 201530 |
|  | Hypoglycemic coma due to type 2 diabetes mellitus | 36714116 |
|  | Polyneuropathy due to type 2 diabetes mellitus | 37017432 |
|  | Gastroparesis due to type 2 diabetes mellitus | 37018728 |
|  | Disorder of nervous system due to type 2 diabetes mellitus | 376065 |
|  | Multiple complications due to type 2 diabetes mellitus | 4099216 |
|  | Arthropathy due to type 2 diabetes mellitus | 4196141 |
|  | Type 2 diabetes mellitus with peripheral angiopathy | 4200875 |
|  | Hyperosmolar non-ketotic state due to type 2 diabetes mellitus | 4215719 |
|  | Persistent microalbuminuria due to type 2 diabetes mellitus | 4221487 |
|  | Cataract due to diabetes mellitus type 2 | 4221495 |
|  | Mononeuropathy due to type 2 diabetes mellitus | 4222415 |
|  | Gangrene due to type 2 diabetes mellitus | 4222876 |
|  | Exudative maculopathy due to type 2 diabetes mellitus | 4223463 |
|  | Persistent proteinuria due to type 2 diabetes mellitus | 4223739 |
|  | Retinopathy due to type 2 diabetes mellitus | 4226121 |
|  | Ketoacidotic coma due to type 2 diabetes mellitus | 4228443 |
|  | Neuropathic arthropathy due to type 2 diabetes mellitus | 43531563 |
|  | Peripheral circulatory disorder due to type 2 diabetes mellitus | 443729 |
|  | Renal disorder due to type 2 diabetes mellitus | 443731 |
|  | Disorder due to type 2 diabetes mellitus | 443732 |
|  | Disorder of eye due to type 2 diabetes mellitus | 443733 |
|  | Ketoacidosis due to type 2 diabetes mellitus | 443734 |
| Valvular heart disease | Valvular heart disease | 40399388 |
|  | Aortic valve stenosis | 4189343 |
|  | Mitral valve regurgitation | 443962 |
|  | Aortic valve regurgitation | 315564 |
|  | Aortic valve stenosis with insufficiency | 4111099 |
|  | Disorders of both mitral and tricuspid valves | 4111566 |
|  | Transplanted heart valve present | 42538119 |
|  | Combined disorders of mitral, aortic and tricuspid valves | 4108235 |
|  | Aortic valve disorder | 314054 |
|  | Tricuspid incompetence, non-rheumatic | 4111412 |
|  | Transplanted heart valve present | 42538119 |
|  | Non-rheumatic mitral valve stenosis with regurgitation | 45766207 |
|  | Non-rheumatic mitral valve disease | 45769461 |
|  | Non-rheumatic heart valve disorder | 4158911 |
|  | Aortic incompetence, non-rheumatic | 4111409 |
|  | Non-rheumatic aortic sclerosis | 4152384 |
|  | Multiple valve disease | 4108234 |
|  | Tricuspid valve disorder, non-rheumatic | 4108817 |

| Ventricular fibrillation | Ventricular fibrillation | 437894 |
| --- | --- | --- |
|  | Ventricular fibrillation and flutter | 4111700 |

Predefined measurements

Note that all descendants of these concepts were also included.

| Measurements | Concept name | Concept ID |
| --- | --- | --- |
| CT of heart with contrast | CT of heart with contrast | 35608046 |
| Cardiac MRI | Cardiac MRI | 4082987 |
|  | MRI for cardiac morphology, function, and velocity | 42710005 |
|  | MRI of heart with contrast | 42537414 |
|  | Magnetic resonance imaging stress study of heart with contrast | 42537586 |
| Cardiac catheterization | Angiography of coronary artery | 4142645 |
|  | Cardiac catheterization | 4223020 |
|  | Cardiac catheterization, left heart, retrograde, percutaneous | 4173410 |
|  | Cardiac catheterization, left heart, transseptal | 4284246 |
|  | Catheterization of right heart | 4223626 |
| Echocardiography | Echocardiography | 4230911 |
|  | Transesophageal echocardiography | 4019824 |
|  | Transthoracic echocardiography | 4335825 |
|  | Ultrasound procedure on cardiovascular system | 4032254 |
| Endomyocardial biopsy | Endomyocardial biopsy | 4308808 |
| Exercise test | Electrocardiogram with exercise test | 4261794 |
|  | Exercise stress echocardiography | 4335824 |
|  | Exercise test finding | 4090324 |
|  | Exercise tolerance test | 4065416 |
| Genetic test | ACTC1 (actin alpha cardiac muscle 1) gene variant measurement | 35952645 |
|  | MYBPC3 (myosin binding protein C3) gene variant measurement | 35957287 |
|  | MYH7 (myosin heavy chain 7) gene variant measurement | 35947206 |
|  | MYL2 (myosin light chain 2) gene variant measurement | 35952884 |
|  | MYL3 (myosin light chain 3) gene variant measurement | 35948676 |
|  | TNNI3 (troponin I3, cardiac type) gene variant measurement | 35947472 |
|  | TNNT2 (troponin T2, cardiac type) gene variant measurement | 35964135 |
|  | TPM1 (tropomyosin 1) gene variant measurement | 35946916 |
| Heart ventricle Left outflow tract | Heart ventricle Left outflow tract | 1031031 |
| Holter electrocardiogram | Holter extended electrocardiographic recording | 4140473 |
|  | Holter monitor test interpretation | 4205487 |
| Left ventricular Ejection fraction | Cardiac ejection fraction | 4323433 |
|  | Left ventricular Ejection fraction | 3027172 |
| Left ventriculogram | Left ventriculogram | 4141016 |
| Maximum left ventricle thickness | Maximum left ventricle thickness | 1989021 |

Predefined medications

Note that all descendants of these concepts were also included. Drug eras with a time gap smaller than 90 days where merged and considered as the same drug era.

| **Concept name** | **Concept ID** |
| --- | --- |
| ACE inhibitors plain | 21601784 |
| Aldosterone antagonists | 21601533 |
| Amiodarone systemic | 21600270 |
| Angiotensin II receptor blockers plain | 21601823 |
| Beta blocking agents | 21601665 |
| Digoxin systemic | 21600234 |
| Direct factor Xa inhibitors | 43534760 |
| Disopyramide systemic | 21600252 |
| Diuretics | 21601461 |
| Heparin group | 21600972 |
| Mavacantem | 779591 |
| Platelet aggregation inhibitors excluding Heparin | 21600985 |
| Selective calcium channel blockers with direct cardiac effects | 21601765 |
| Vitamin K antagonists | 21600962 |
| enoxaparin | 1301025 |

Predefined procedures

Note that all descendants of these concepts were also included. The following concepts were excluded:

- For cardiac pacemaker procedure: Complete removal of implanted cardiac pacemaker system (4019140), Removal of implanted cardiac pacemaker system or components (4050575), and Removal of cardioverter/defibrillator pulse generator without replacement (4228672)
- For cardioverter defibrillator procedure: External ventricular defibrillation (4138468), Removal of internal cardiac defibrillator (4216485), Removal of subcutaneous cardioverter defibrillator (44810459)

| Procedures | Concept name | Concept ID |
| --- | --- | --- |
| Cardiac pacemaker procedure | Cardiac pacemaker procedure | 4051938 |
|  | Implantation of cardiac pacemaker | 4144921 |
|  | Indirect temporary cardiac pacemaker procedure | 4049400 |
|  | Insertion of endocardial electrode for temporary cardiac pacing | 4051939 |
|  | Introduction of cardiac pacemaker system via vein | 4019139 |
|  | Percutaneous transcatheter septal reduction therapy (eg, alcohol septal ablation) including temporary pacemaker insertion when performed | 44816449 |
| Cardioverter defibrillator procedure | Cardioverter defibrillator procedure | 4138751 |
|  | Implantation of internal cardiac defibrillator | 4191948 |
| Screening for cardiovascular system disease | Screening for cardiovascular system disease | 4102739 |
| Seen by specialist or cardiologist | Letter from consultant | 4078131 |
|  | Letter from specialist | 4151573 |
|  | Referral to cardiologist | 4204664 |
|  | Referral to cardiology service | 4080077 |
|  | Seen by cardiologist | 4139722 |
|  | Seen in cardiac clinic | 4083416 |
| Septal reduction therapy | Non-surgical septal reduction therapy (eg, alcohol ablation), for hypertrophic obstructive cardiomyopathy; with coronary arteriograms, with or without temporary pacemaker (Deprecated) | 42739918 |
|  | Nonsurgical reduction of cardiac septum for hypertrophic obstructive cardiomyopathy with angiography of coronary artery | 4260103 |
|  | Percutaneous transcatheter septal reduction therapy (eg, alcohol septal ablation) including temporary pacemaker insertion when performed | 44816449 |
|  | Percutaneous transluminal ablation of septum of heart using chemical substance | 40483085 |
|  | Percutaneous transluminal septal myocardial ablation | 4229994 |
|  | Septal myectomy | 37397338 |
|  | Ventricular septal myectomy | 45765641 |
| Septal reduction therapy non surgical | Non-surgical septal reduction therapy (eg, alcohol ablation), for hypertrophic obstructive cardiomyopathy; with coronary arteriograms, with or without temporary pacemaker | 42739918 |
|  | Nonsurgical reduction of cardiac septum for hypertrophic obstructive cardiomyopathy with angiography of coronary artery | 4260103 |
|  | Percutaneous transcatheter septal reduction therapy (eg, alcohol septal ablation) including temporary pacemaker insertion when performed | 44816449 |
| Septal reduction therapy surgical | Percutaneous transluminal ablation of septum of heart using chemical substance | 40483085 |
|  | Percutaneous transluminal septal myocardial ablation | 4229994 |
|  | Septal myectomy | 37397338 |
|  | Ventricular septal myectomy | 45765641 |
| Transplantation of heart | Transplantation of heart | 4137127 |
|  | Transplanted heart present | 42538117 |
| Transplanted heart valve present | Transplanted heart valve present | 42538119 |

# **Supplementary Note 2.** Direct Age Standardisation for clinically recognised HCM prevalence

To calculate directly age-standardised prevalence rates of clinically recognised hypertrophic cardiomyopathy (HCM), we applied the age-specific prevalence estimates from our study population to the age distribution of the World Health Organization (WHO) world standard population (i.e., how much of the total world population falls into that age group, according to WHO's reference standard: <https://seer.cancer.gov/stdpopulations/stdpop.singleages.html>). We used the following age categories: 18 to 29, 30 to 39, 40 to 49, 50 to 59, 60 to 60, 70 to 79, and 80+.

The age-standardised prevalence was calculated by multiplying each age-specific prevalence by the corresponding WHO world standard population weight and summing the weighted values across all age groups.

# **Supplementary Note 3.** Patient-level characterisation stratified by sex results.

All results are shown in the [interactive web application](https://dpa-pde-oxford.shinyapps.io/HCM_Characterisation/)

*Comorbidities*

In the clinically recognised HCM cohort, any time prior to index date women showed higher rates of valvular heart disease compared to men (27% in females vs 19% in males in SIDIAP, 26% vs 18% in NLHR). Essential hypertension was also reported more frequently in women (62% vs 54% in NLHR, 86% vs 79% in NAJS), as well as chronic kidney disease (16% vs 8% in CPRD GOLD). On the other hand, men showed higher rates of chronic obstructive pulmonary disease (11% vs 19% in SIDIAP).

In the clinically recognised oHCM cohort, sex-frequency differences were slightly higher for chronic kidney disease (18% in females vs 6% in males in CPRD GOLD), essential hypertension (67% vs 57% in NLHR, 61% vs 52% in SIDIAP), and valvular heart disease (29% vs 19% in SIDIAP, 32% vs 24% in NLHR). Disorder of lipoprotein and/or lipid metabolism was also reported more frequently in females (48% vs 40% in SIDIAP, 26% vs 18% in NLHR). Chronic obstructive pulmonary disease sex-differences where not as pronounced within the clinically recognised oHCM cohort (10% vs 14%), but sex-differences in frequencies of ischaemic heart disease and cardiac arrhythmia increased in NLHR (26% vs 34%, 33% vs 41%, respectively).

*Diagnostic measurements*

In the clinically recognised HCM cohort there were no notable differences among females and males, although cardiac catheterization and cardiac MRI were more frequent in males compared to women three months prior the index date (31% vs 36% and 10% vs 14% in InGef RDB, respectively).

In the clinically recognised oHCM cohort, Holter electrocardiogram was double more frequent in males compared to women (4% vs 8% in NAJS) three months prior the index date. Differences on cardiac catheterization and cardiac MRI in InGef RDB were less pronounced than in the clinically recognised HCM cohort (31% vs 35% and 9% vs 12%).

*Medications*

In the clinically recognised HCM cohort, diuretics were more frequent among females than in males any time prior to index date (49% vs 28% in CPRD GOLD, 69% vs 53% in SIDIAP, 58% vs 48% in NAJS, 55% vs 46 in InGef RDB, and 37% vs 29% in DK-DHR). These differences remained significant three months prior to index date (33% vs 18% in CPRD GOLD, 49% vs 38% in SIDIAP) and three months after the index date (33% vs 20% in CPRD GOLD, 54% vs 43% in SIDIAP). Angiotensin II receptor blockers were also more frequently prescribed to women (37% vs 28% in SIDIAP, 19% vs 13% in CPRD GOLD) any time prior the index date, although differences were smaller three months prior the index date (17% vs 13% in SIDIAP, 13 vs 9% in CPRD GOLD) and after three months of the diagnosis (18% vs 14% in SIDIAP, 13% vs 10% in CPRD GOLD).

Among the clinically recognised oHCM cohort, sex-differences among diuretics prescriptions any time prior the index date were also significant (51% vs 28% in CPRD GOLD, 64% vs 46% in SIDIAP, 54% vs 41% in InGef RDB, 38% vs 29% in DK-DHR). Angiotensin II receptor blockers also remained more frequent in women (35% vs 26% in SIDIAP, 20% vs 13% in CPRD GOLD). Interestingly, aldosterone antagonists prescription frequency was higher in men compared to women in NAJS, either three months prior to index date (9% vs 18%) and three months after the index date (17% to 27%).

*Treatment procedures*

There were no notable differences among females and males.

# **Supplementary Note 4.** Time from the first record of comorbidities, measurements, and medications to index date

All results are shown in the [interactive web application](https://dpa-pde-oxford.shinyapps.io/HCM_Characterisation/)*.*

In the clinically recognised HCM cohort, median time since first comorbidity, measurement, and medication recorded to the diagnosis day was more than one year. However, some exceptions include ventricular fibrillation, with a median time of -242 days to -233 days (CPRD GOLD, SIDIAP – InGef RDB). Additionally, InGef RDB differed from other databases in that most measurements were first recorded within the month before the index date.

Overall, similar timing patterns were observed between females and males for most of the covariates. However, there were some differences between the median time from the first diagnosis of some comorbidities and the time of the recorded HCM diagnosis. In DK-DHR database, median time to first diagnosis of aberrant premature complexes to clinically recognised HCM was nearly 7 years in women and 5 years in male. In CPRD GOLD, chronic obstructive pulmonary disease was typically diagnosed earlier in females (5 years before the recorded HCM diagnosis) than in males (about 3 years), as well as type 2 diabetes mellitus (approximately 7 years vs 4.5 years), and essential hypertension (8.5 years vs 6.5 years). In contracts, obesity and coronary arteriosclerosis were recorded earlier in males, with nearly 9 years vs. 6 years for obesity (CPRD GOLD), and over 9 years vs nearly 7 years for coronary arteriosclerosis (DK-DHR).

Cardiac catheterization was also earlier measured in women (5 years) compared to men (2.5 years) in CPRD GOLD.

In DK-DHR, diuretics, selective calcium channel blockers with direct cardiac effects, aldosterone antagonists, and amiodarone systemic, were shown to be earlier prescribed to women compared to men (13 years vs 8 years, 12 vs 8, 5 vs 2.5, and 4 vs 2, respectively). In CPRD GOLD, the diuretics trend was also seen (8 vs 5), and additional differences in digoxin systemic and ACE inhibitors (5 vs 3, 7 vs 5).

Similarly, in the clinically recognised oHCM cohort, most comorbidities, measurements, and medications were also first recorded more than one year before the index date, except for disopyramide treatment in SIDIAP, with a median time of -306 days (IQR: -935 to -78).

Generally, there were no notable differences between females and males in terms of the time from first recorded covariate to recorded oHCM diagnosis. However, median time to first diagnosis of aberrant premature complexes to clinically recognised oHCM was also earlier in females than in males (4 vs 2) in NLHR, as well as type 2 diabetes mellitus (8 vs 6) in DK-DHR. Atrioventricular block and ischaemic stroke were also found to be reported earlier in women than in men (6 vs 3.5, DK-DHR; 4.5 vs 2, CPRD GOLD, respectively). Coronary arteriosclerosis remained to be recorded earlier in males (almost a year vs 3, CPRD GOLD). Obesity trend was mixed, showing an earlier diagnosis for males in CPRD GOLD (5 vs 8), and an earlier record for females in NLHR (6 vs 4).

Echocardiography measurement was found to be earlier recorded in females than in men (3.5 vs 1) in CPRD GOLD).

Diuretics were also prescribed earlier to females than male (13 vs 7 in DKR-DHR, 8 vs 4 in CPRD GOLD), as were selective calcium channel blockers with direct cardiac effects (11 vs 6 in DK-DHR). In CPRD GOLD, heparins and angiotensin II receptor blockers were also prescribed earlier to females (5.5 vs 1, 7 vs 4.5). Conversely, amiodarone systemic was prescribed earlier to males (2 vs 4), while digoxin systemic showed mixed trends – earlier in females in CPRD GOLD (5.5 vs 3), whereas earlier in males in NLHR (1.5 vs 4).

# **Supplementary Table 1.** Description of the databases.

| **Database** | **Country** | **Healthcare setting** | **Type of data** | **Start date** | **End date** |
| --- | --- | --- | --- | --- | --- |
| CPRD-GOLD | United Kingdom | Primary care | Electronic health records | 01/01/2010 | 15/06/2024 |
| DK-DHR | Denmark | Primary care, community pharmacists, secondary care (specialists, hospital inpatient care) | Registry | 01/01/2010 | 15/03/2024 |
| InGef RDB | Germany | Primary care, community pharmacists, secondary care (specialists, hospital inpatient care) | Claims | 01/01/2015 | 30/09/2024 |
| NAJS | Croatia | Primary care, secondary care (specialists, hospital inpatient data) | Claims | 01/01/2014 | 30/01/2025 |
| NLHR | Norway | Primary care, secondary care (specialists, hospital inpatient care) | Registry | 01/01/2010 | 31/12/2023 |
| SIDIAP | Spain | Primary care with hospital linkage | Electronic health records | 01/01/2010 | 30/06/2023 |

| **Variables** | **Levels** | **Statistics** | **Database (Country)** | | | | | | | | | | | |
| --- | --- | --- | --- | --- | --- | --- | --- | --- | --- | --- | --- | --- | --- | --- |
|  |  |  | **CPRD GOLD (UK)** | | **DK-DHR (Denmark)** | | **InGef RDB (Germany)** | | **NAJS (Croatia)** | | **NLHR (Norway)** | | **SIDIAP (Spain)** | |
|  |  |  | **Cohort name** | | | | | | | | | | | |
|  |  |  | **HCM** | **oHCM** | **HCM** | **oHCM** | **HCM** | **oHCM** | **HCM** | **oHCM** | **HCM** | **oHCM** | **HCM** | **oHCM** |
| **Baseline characteristics** | | | | | | | | | | | | | | |
| Number of records | - | N | 1,780 | 1,062 | 4,826 | 1,934 | 6,311 | 2,551 | 6,653 | 1,559 | 7,114 | 2,413 | 13,593 | 2,844 |
| Number of subjects | - | N | 1,780 | 1,062 | 4,826 | 1,934 | 6,311 | 2,551 | 6,653 | 1,559 | 7,114 | 2,413 | 13,593 | 2,844 |
| Age (years) | - | Median [Q25-Q75] | 61 [50-71] | 61 [50-71] | 67 [56-77] | 69 [60-77] | 66 [54-78] | 66 [55-78] | 71 [60-79] | 72 [63-80] | 66 [54-75] | 67 [58-75] | 73 [61-82] | 72 [61-81] |
|  |  | Range [Min-Max] | 18-95 | 18-95 | 18-101 | 18-99 | 18-103 | 18-100 | 18-103 | 19-100 | 18-99 | 18-97 | 18-101 | 18-101 |
| Sex | Female | N (%) | 663 (37) | 417 (39) | 2,272 (47) | 1,077 (56) | 2,431 (39) | 1,167 (46) | 3,170 (48) | 772 (50) | 2,861 (40) | 1,163 (48) | 6,159 (45) | 1,620 (57) |
|  | Male | N (%) | 1,117 (63) | 645 (61) | 2,554 (53) | 857 (44) | 3,880 (61) | 1,384 (54) | 3,483 (52) | 787 (50) | 4,253 (60) | 1,250 (52) | 7,434 (55) | 1,224 (43) |
| **Comorbidities any time prior first recorded HCM or oHCM recorded diagnosis** | | | | | | | | | | | | | | |
| Aberrant premature complexes | - | N (%) | 0 (0) | 0 (0) | 118 (2) | 46 (2) | 144 (2) | 49 (2) | 181 (3) | 32 (2) | 212 (3) | 49 (2) | 400 (3) | 60 (2) |
| Atrial fibrillation | - | N (%) | 191 (11) | 111 (10) | 241 (5) | 73 (4) | 1,194 (19) | 354 (14) | 642 (10) | 177 (11) | 850 (12) | 216 (9) | 3,325 (24) | 525 (18) |
| Atrioventricular block | - | N (%) | 7 (0) | 5 (0) | 177 (4) | 37 (2) | 260 (4) | 92 (4) | 178 (3) | 31 (2) | 337 (5) | 96 (4) | 790 (6) | 144 (5) |
| Cardiac arrhythmia | - | N (%) | 282 (16) | 164 (15) | 1,235 (26) | 409 (21) | 1,743 (28) | 564 (22) | 2,579 (39) | 641 (41) | 2,845 (40) | 894 (37) | 5,681 (42) | 983 (35) |
| Chronic kidney disease | - | N (%) | 197 (11) | 114 (11) | 217 (4) | 58 (3) | 1,066 (17) | 330 (13) | 700 (11) | 180 (12) | 380 (5) | 136 (6) | 3,327 (24) | 566 (20) |
| Chronic obstructive pulmonary disease | - | N (%) | 92 (5) | 62 (6) | 366 (8) | 158 (8) | 494 (8) | 190 (7) | 1,113 (17) | 314 (20) | 681 (10) | 250 (10) | 2,075 (15) | 327 (11) |
| Coronary arteriosclerosis | - | N (%) | 34 (2) | 16 (2) | 286 (6) | 111 (6) | 1,340 (21) | 463 (18) | 376 (6) | 149 (10) | 849 (12) | 299 (12) | 907 (7) | 155 (5) |
| Disorder of lipoprotein and or lipid metabolism | - | N (%) | 198 (11) | 119 (11) | 754 (16) | 299 (15) | 1,574 (25) | 558 (22) | 2,711 (41) | 657 (42) | 1,481 (21) | 527 (22) | 6,177 (45) | 1,268 (45) |
| Essential hypertension | - | N (%) | 555 (31) | 344 (32) | 93 (2) | 28 (1) | 3,081 (49) | 1,151 (45) | 5,487 (82) | 1,345 (86) | 4,067 (57) | 1,483 (61) | 8,101 (60) | 1,619 (57) |
| Heart failure | - | N (%) | 79 (4) | 46 (4) | 669 (14) | 210 (11) | 1,623 (26) | 522 (20) | 1,368 (21) | 361 (23) | 1,695 (24) | 503 (21) | 4,051 (30) | 632 (22) |
| Ischemic stroke | - | N (%) | 84 (5) | 56 (5) | 474 (10) | 176 (9) | 450 (7) | 165 (6) | 601 (9) | 172 (11) | 796 (11) | 272 (11) | 1,374 (10) | 241 (8) |
| Ischemic heart disease | - | N (%) | 258 (14) | 161 (15) | 1,253 (26) | 508 (26) | 1,763 (28) | 590 (23) | 2,136 (32) | 570 (37) | 2,103 (30) | 724 (30) | 2,950 (22) | 530 (19) |
| Obesity | - | N (%) | 54 (3) | 35 (3) | 368 (8) | 148 (8) | 778 (12) | 272 (11) | 746 (11) | 186 (12) | 501 (7) | 157 (7) | 3,826 (28) | 783 (28) |
| Premature atrial contraction | - | N (%) | 0 (0) | 0 (0) | 0 (0) | 0 (0) | 0 (0) | 0 (0) | 0 (0) | 0 (0) | 0 (0) | 0 (0) | 0 (0) | 0 (0) |
| Sudden cardiac death | - | N (%) | 0 (0) | 0 (0) | 0 (0) | 0 (0) | <5 | 0 (0) | <5 | 0 (0) | 0 (0) | 0 (0) | 0 (0) | 0 (0) |
| Sustained ventricular tachycardia | - | N (%) | 13 (1) | 5 (0) | 101 (2) | 30 (2) | 162 (3) | 36 (1) | 125 (2) | 40 (3) | 293 (4) | 81 (3) | 182 (1) | 23 (1) |
| Type 2 diabetes mellitus | - | N (%) | 159 (9) | 101 (10) | 490 (10) | 182 (9) | 1,004 (16) | 339 (13) | 2,087 (31) | 506 (32) | 747 (11) | 270 (11) | 3,750 (28) | 632 (22) |
| Valvular heart disease | - | N (%) | 112 (6) | 66 (6) | 628 (13) | 379 (20) | 927 (15) | 361 (14) | 947 (14) | 299 (19) | 1,515 (21) | 673 (28) | 3,067 (23) | 699 (25) |
| Ventricular fibrillation | - | N (%) | <5 | <5 | 30 (1) | 9 (0) | 30 (0) | 7 (0) | 44 (1) | 16 (1) | 53 (1) | 12 (0) | 30 (0) | 6 (0) |

**Supplementary Table 2.** Annual period prevalence (95% confidence intervals -CI-) of clinically recognised hypertrophic cardiomyopathy (HCM) and obstructive HCM (oHCM) by study database

| **Prevalence start date** | **Prevalence end date** | **CPRD GOLD (UK)** | **DK-DHR (Denmark)** | **InGef RDB (Germany)** | **NAJS (Croatia)** | **NLHR (Norway)** | **SIDIAP (Spain)** |
| --- | --- | --- | --- | --- | --- | --- | --- |
| **HCM** | | | | | | | |
| 2010-01-01 | 2010-12-31 | 0.022 (0.0208, 0.0232) | 0.0601 (0.0578, 0.0625) | - | - | 0.0407 (0.0386, 0.0429) | 0.1491 (0.1458, 0.1526) |
| 2011-01-01 | 2011-12-31 | 0.0238 (0.0225, 0.0251) | 0.0638 (0.0614, 0.0662) | - | - | 0.05 (0.0477, 0.0524) | 0.1557 (0.1523, 0.1592) |
| 2012-01-01 | 2012-12-31 | 0.0261 (0.0248, 0.0275) | 0.0691 (0.0666, 0.0716) | - | - | 0.0572 (0.0547, 0.0597) | 0.1635 (0.16, 0.1671) |
| 2013-01-01 | 2013-12-31 | 0.0276 (0.0262, 0.0291) | 0.0729 (0.0704, 0.0755) | - | - | 0.0652 (0.0626, 0.0678) | 0.1702 (0.1666, 0.1739) |
| 2014-01-01 | 2014-12-31 | 0.0297 (0.0282, 0.0312) | 0.0759 (0.0734, 0.0786) | - | - | 0.0734 (0.0707, 0.0762) | 0.1877 (0.1839, 0.1916) |
| 2015-01-01 | 2015-12-31 | 0.0313 (0.0298, 0.033) | 0.0792 (0.0765, 0.0819) | - | - | 0.0824 (0.0796, 0.0853) | 0.2062 (0.2022, 0.2103) |
| 2016-01-01 | 2016-12-31 | 0.0334 (0.0316, 0.0352) | 0.0826 (0.08, 0.0854) | 0.0278 (0.0265, 0.0291) | 0.0958 (0.0925, 0.0993) | 0.0926 (0.0897, 0.0956) | 0.2178 (0.2137, 0.222) |
| 2017-01-01 | 2017-12-31 | 0.0353 (0.0334, 0.0373) | 0.0858 (0.0831, 0.0886) | 0.0366 (0.0351, 0.0381) | 0.1112 (0.1077, 0.1148) | 0.1032 (0.1002, 0.1064) | 0.2294 (0.2251, 0.2337) |
| 2018-01-01 | 2018-12-31 | 0.0367 (0.0347, 0.0388) | 0.0871 (0.0844, 0.0899) | 0.044 (0.0424, 0.0457) | 0.1196 (0.116, 0.1234) | 0.1138 (0.1107, 0.1171) | 0.2352 (0.2309, 0.2395) |
| 2019-01-01 | 2019-12-31 | 0.0378 (0.0357, 0.04) | 0.0906 (0.0878, 0.0935) | 0.0511 (0.0494, 0.0529) | 0.1289 (0.1252, 0.1327) | 0.1231 (0.1198, 0.1265) | 0.2383 (0.2341, 0.2427) |
| 2020-01-01 | 2020-12-31 | 0.0391 (0.0369, 0.0414) | 0.0953 (0.0924, 0.0982) | 0.0573 (0.0555, 0.0592) | 0.1346 (0.1308, 0.1385) | 0.1311 (0.1277, 0.1345) | 0.2352 (0.2309, 0.2395) |
| 2021-01-01 | 2021-12-31 | 0.0411 (0.0388, 0.0435) | 0.0972 (0.0943, 0.1001) | 0.0637 (0.0618, 0.0657) | 0.1391 (0.1353, 0.1431) | 0.1387 (0.1353, 0.1423) | 0.2343 (0.2301, 0.2386) |
| 2022-01-01 | 2022-12-31 | 0.0422 (0.0398, 0.0448) | 0.1003 (0.0974, 0.1033) | 0.0701 (0.068, 0.0722) | 0.1422 (0.1383, 0.1461) | 0.1499 (0.1464, 0.1536) | 0.2367 (0.2325, 0.241) |
| 2023-01-01 | 2023-12-31 | 0.0431 (0.0406, 0.0458) | 0.1025 (0.0995, 0.1055) | 0.0759 (0.0738, 0.0781) | 0.1484 (0.1445, 0.1525) | 0.1598 (0.1562, 0.1636) |  |
| 2024-01-01 | 2024-12-31 |  |  |  | 0.1547 (0.1507, 0.1589) |  |  |
| **oHCM** | | | | | | | |
| 2010-01-01 | 2010-12-31 | 0.0163 (0.0153, 0.0174) | 0.0342 (0.0325, 0.036) |  |  | 0.0226 (0.021, 0.0242) | 0.022 (0.0207, 0.0233) |
| 2011-01-01 | 2011-12-31 | 0.0176 (0.0165, 0.0188) | 0.0361 (0.0343, 0.0379) |  |  | 0.0264 (0.0248, 0.0282) | 0.0218 (0.0206, 0.0231) |
| 2012-01-01 | 2012-12-31 | 0.0193 (0.0182, 0.0205) | 0.0382 (0.0364, 0.0401) |  |  | 0.0298 (0.028, 0.0316) | 0.0218 (0.0206, 0.0232) |
| 2013-01-01 | 2013-12-31 | 0.0202 (0.019, 0.0214) | 0.0396 (0.0377, 0.0415) |  |  | 0.0333 (0.0315, 0.0352) | 0.025 (0.0236, 0.0264) |
| 2014-01-01 | 2014-12-31 | 0.0215 (0.0202, 0.0228) | 0.0412 (0.0393, 0.0432) |  |  | 0.037 (0.0351, 0.0389) | 0.0336 (0.0321, 0.0353) |
| 2015-01-01 | 2015-12-31 | 0.0226 (0.0213, 0.0241) | 0.0424 (0.0405, 0.0444) |  |  | 0.0412 (0.0392, 0.0432) | 0.0406 (0.0388, 0.0424) |
| 2016-01-01 | 2016-12-31 | 0.0235 (0.022, 0.025) | 0.0438 (0.0419, 0.0458) | 0.0134 (0.0125, 0.0143) | 0.0203 (0.0188, 0.0219) | 0.0455 (0.0435, 0.0477) | 0.0447 (0.0429, 0.0467) |
| 2017-01-01 | 2017-12-31 | 0.0242 (0.0227, 0.0259) | 0.0448 (0.0428, 0.0468) | 0.0175 (0.0165, 0.0186) | 0.0259 (0.0242, 0.0277) | 0.0504 (0.0483, 0.0526) | 0.0491 (0.0472, 0.0511) |
| 2018-01-01 | 2018-12-31 | 0.0249 (0.0233, 0.0267) | 0.0451 (0.0432, 0.0471) | 0.021 (0.0199, 0.0222) | 0.0279 (0.0262, 0.0297) | 0.0549 (0.0528, 0.0572) | 0.0532 (0.0512, 0.0553) |
| 2019-01-01 | 2019-12-31 | 0.0248 (0.0231, 0.0265) | 0.0462 (0.0443, 0.0483) | 0.0247 (0.0235, 0.0259) | 0.0289 (0.0272, 0.0308) | 0.0582 (0.056, 0.0606) | 0.0553 (0.0532, 0.0574) |
| 2020-01-01 | 2020-12-31 | 0.025 (0.0233, 0.0268) | 0.0483 (0.0462, 0.0504) | 0.0274 (0.0261, 0.0287) | 0.0312 (0.0294, 0.0331) | 0.061 (0.0587, 0.0634) | 0.0556 (0.0536, 0.0577) |
| 2021-01-01 | 2021-12-31 | 0.026 (0.0242, 0.028) | 0.0488 (0.0468, 0.0509) | 0.0305 (0.0291, 0.0319) | 0.0322 (0.0304, 0.0341) | 0.0635 (0.0612, 0.0659) | 0.0569 (0.0548, 0.059) |
| 2022-01-01 | 2022-12-31 | 0.0265 (0.0246, 0.0286) | 0.0499 (0.0478, 0.052) | 0.0328 (0.0314, 0.0342) | 0.0329 (0.0311, 0.0349) | 0.0666 (0.0642, 0.0691) | 0.0591 (0.057, 0.0613) |
| 2023-01-01 | 2023-12-31 | 0.0266 (0.0246, 0.0287) | 0.05 (0.048, 0.0521) | 0.0357 (0.0342, 0.0372) | 0.0339 (0.032, 0.0359) | 0.0691 (0.0667, 0.0716) |  |
| 2024-01-01 | 2024-12-31 |  |  |  | 0.0344 (0.0326, 0.0364) |  |  |

# **Supplementary Figure 1.** Study design diagram.

^a^ Follow-up until the earliest of: end of follow-up, end of data availability or death.

**Note:** Square brackets (“[“ or “]”) indicate that the day is included, while parenthesis (“(“ or “)”) indicates that the day is excluded.

Abbreviations: HCM (Hypertrophic cardiomyopathy), oHCM (obstructive HCM).

# **Supplementary Figure 2.** Attrition flowchart of clinically recognised hypertrophic cardiomyopathy (HCM) and obstructive HCM (oHCM) cohorts by study database. **Note:** Initial quality control steps included: (1) retaining only those records with a start date prior to end date; (2) ensuring records occurred during individual’s observation period; and (3) merging overlapping diagnosis records.

**Supplementary Figure 3.** Annual age-standardised prevalence and 95%CI of the first record of clinically recognised HCM and oHCM cohorts by study database. All point estimates, as well as 95%CI, can be found in the [interactive web application](https://data-dev.darwin-eu.org/HCM_Characterisation_Manuscript/)

**Supplementary Figure 4.** Frequency (%) of low-recorded comorbidities by study database and time window (in days). In panels the frequency of the comorbidities is shown for the clinically recognised (A) HCM cohort, and (B) oHCM cohort. All time windows and exact frequencies can be found in the [interactive web application](https://dpa-pde-oxford.shinyapps.io/HCM_Characterisation/).

**Supplementary Figure 5.** Frequency (%) of low-recorded medications by study database and time window (in days). In panels the frequency of the comorbidities is shown for the clinically recognised (A) HCM cohort, and (B) oHCM cohort. All time windows and exact frequencies can be found in the [interactive web application](https://dpa-pde-oxford.shinyapps.io/HCM_Characterisation/).
